# Translation Fidelity and Respiration Deficits in CLPP-Deficient Tissues: Mechanistic Insights from Mitochondrial Complexome Profiling

**DOI:** 10.3390/ijms242417503

**Published:** 2023-12-15

**Authors:** Jana Key, Suzana Gispert, Gabriele Koepf, Julia Steinhoff-Wagner, Marina Reichlmeir, Georg Auburger

**Affiliations:** 1 Goethe University Frankfurt, University Hospital, Clinic of Neurology, Exp. Neurology, Heinrich Hoffmann Str. 7, 60590 Frankfurt am Main, Germany; gispert-sanchez@em.uni-frankfurt.de (S.G.); marina.reichlmeir@kgu.de (M.R.); auburger@em.uni-frankfurt.de (G.A.); 2 TUM School of Life Sciences, Animal Nutrition and Metabolism, Technical University of Munich, Liesel-Beckmann-Str. 2, 85354 Freising-Weihenstephan, Germany; jsw@tum.de

**Keywords:** primary ovarian insufficiency, azoospermia, sensorineural hearing loss, mtLSU quality control, rRNA chaperone ERAL1

## Abstract

The mitochondrial matrix peptidase CLPP is crucial during cell stress. Its loss causes Perrault syndrome type 3 (PRLTS3) with infertility, neurodegeneration, and a growth deficit. Its target proteins are disaggregated by CLPX, which also regulates heme biosynthesis via unfolding ALAS enzymes, providing access for pyridoxal-5′-phosphate (PLP). Despite efforts in diverse organisms with multiple techniques, CLPXP substrates remain controversial. Here, avoiding recombinant overexpression, we employed complexomics in mitochondria from three mouse tissues to identify endogenous targets. A CLPP absence caused the accumulation and dispersion of CLPX-VWA8 as AAA+ unfoldases, and of PLPBP. Similar changes and CLPX-VWA8 co-migration were evident for mitoribosomal central protuberance clusters, translation factors like GFM1-HARS2, the RNA granule components LRPPRC-SLIRP, and enzymes OAT-ALDH18A1. Mitochondrially translated proteins in testes showed reductions to <30% for MTCO1-3, the mis-assembly of the complex IV supercomplex, and accumulated metal-binding assembly factors COX15-SFXN4. Indeed, heavy metal levels were increased for iron, molybdenum, cobalt, and manganese. RT-qPCR showed compensatory downregulation only for *Clpx* mRNA; most accumulated proteins appeared transcriptionally upregulated. Immunoblots validated VWA8, MRPL38, MRPL18, GFM1, and OAT accumulation. Co-immunoprecipitation confirmed CLPX binding to MRPL38, GFM1, and OAT, so excess CLPX and PLP may affect their activity. Our data mechanistically elucidate the mitochondrial translation fidelity deficits which underlie progressive hearing impairment in PRLTS3.

## 1. Introduction

LONP1 and CLPXP are the two soluble proteases of the mammalian mitochondrial matrix, with extreme conservation until bacteria and plant chloroplasts [1,2]. LONP1 is essential for life [3] and plays a crucial role in protein turnover [4]. This protein contains both the AAA+ unfoldase and the peptidase domain necessary for proteolysis [4,5]. LONP1 was shown to assemble into homohexameric rings upon overexpression, purification, and electron microscopy analysis. In this multimer conformation LONP1 achieves maximal activity [6]. In comparison, CLPP absence may even extend the lifespan in eukaryotes [7]; its functions become necessary only in periods of cell stress [1], when it acts in response to stalled ribosomes and possibly in the unfolded protein response of mitochondria (UPRmt) [8,9]. CLPP by itself exerts only a chymotrypsin-like activity towards small peptides. It depends on interactions with the AAA+ unfoldase/disaggregase CLPX in a barrel shape, composed of homomultimeric rings, to enable substrate recognition and unfolding, as well as the activation of its proteolytic capacity [10,11]. Bacterial ClpX was shown to perform chaperone roles independent from ClpP, cooperating with the Hsp70 family of chaperones (DnaJ/DnaK/GroEL) in the HssR defense system against heme–metal toxicity [11,12,13,14,15].

Mutations in human CLPP cause Perrault syndrome type 3 (PRLTS3), an autosomal recessive disorder characterized by complete infertility due to female primary ovarian insufficiency and male azoospermia after meiotic arrest at diplotene, with subsequent sensorineural deafness, followed by insidious neurodegeneration manifesting as ataxia, leukodystrophy, and neuropathy [16,17]. Most variants of Perrault syndrome are triggered by the failure of mitochondrial RNA processing and translation, as exemplified by other causal mutations in the tRNA-amino acid synthases HARS2 and LARS2, in the mitoribosomal factor RMND1, in the RNA degradation factor PRORP, in the nucleoid replication/repair factor TWNK, or in CLPP [18,19].

In contrast, mutations in human CLPX trigger hematological disorders due to errors in heme biosynthesis [20,21]. In zebrafish, the knockdown of CLPX can be rescued through the supplementation of the heme precursor ALA (delta-amino levulinic acid) [22]. A common iron/heme-related pathway connects the two diverse clinical manifestations, given that porphyrias can progress into ataxia/leukodystrophy [23,24,25,26,27,28,29]. The relevant CLPX functions independent from CLPP were uncovered in *Saccharomyces cerevisiae* (where CLPP is not conserved) via a genetic screen, which showed that the CLPX ortholog activates the heme biosynthesis enzyme ALAS (5-aminolevulinic acid synthase), so CLPX deletion causes a 5-fold reduction in ALA and a 2-fold reduction in heme [30]. This study also reported that CLPX achieves this activation by controlling the incorporation of its cofactor, pyridoxal-5′-phosphate (PLP), into ALAS. PLP is a vitamin B6 derivative, which mediates Schiff base formation after a nucleophilic attack by lysine within the enzyme active site, but also has a chaperone role in protein folding [31]. This CLPX role for PLP access to ALAS and the folding control of this enzyme is conserved until mammals [30]. Cells modulate the toxicity of excess porphyrin intermediates through the very tight regulation of ALAS expression, with dependence on iron availability. The completion of heme synthesis is governed further downstream again in mitochondria via the CLPX modification of protoporphyrin IX oxidase (PPOX) activity and ferrochelatase (FECH) levels. For chlorophyll biosynthesis, plant cells also have feedback from ALA levels onto the crucial porphyrin activation by magnesium chelatase [32,33]. Plant magnesium chelatase protein sequences show the coexistence of the AAA+ and VWA domains, which appeared first in *Synechocystis* cyanobacteria within the catalytic ChlD subunit and is still conserved in the mitochondrial VWA8 unfoldase of mammals despite their lack of chlorophyll production [34].

Bacterial ClpP can also interact with other AAA+ unfoldases from the Hsp100 family such as ClpA [35,36], but in mammals, only the AAA+ unfoldase CLPB remains conserved, and no stable interaction has been observed between ClpX and ClpB in bacteria [36]. AAA+ domains typically adapt a homohexamer conformation for maximal efficiency, whereas ClpP assembles into homoheptameric rings [37,38], and a fully assembled ClpXP degradation machine in *E. coli* was shown to adopt a barrel-like form: two ClpP rings in the center represent the proteolytic chamber, while ClpX rings on either side are responsible for the targeting and linearization of unfolded or aggregated substrates [39]. The central cavity within the hexameric AAA+ ring can provide a tunnel through which RNA or DNA can be threaded to alter the assembly or remove associated proteins [1,34]. As the first question of this study, we investigate whether mammalian AAA+ unfoldases other than CLPX interact physically or functionally with CLPP within the same folding pathways. Perhaps another AAA+ unfoldase assumes the workload when the CLPXP apparatus is dysfunctional. There are several candidates with known roles for mitochondrial protein complex dynamics and degradation in other contexts. They include SPATA5 and VWA8 (presumably, given its BIOGRID interaction profile) in mitoribosomal and respiratory chain remodeling [40], BCS1L in respiratory complex III assembly [41], YME1L1, AFG3L2 and SPG7 in the mitochondrial inner membrane [42], and VCP and ATAD3 exerting unfolded protein response (UPR) tasks in the membranes of the endoplasmic reticulum and mitochondria [43,44].

As main targets of the CLPXP degradation machinery in bacteria and mammalian mitochondria during cell stress, incomplete translation products at stalled mitoribosomes are thought to be disaggregated and degraded. It was demonstrated that a CLPP deficiency in mice causes impaired translation fidelity at mitoribosomes, claiming that this occurs due to the accumulation and inappropriate interactions of the rRNA chaperone ERAL1, which acts mainly in the assembly of mtSSU, the small mitoribosomal subunit [45,46,47]. However, the crucial role of ERAL1 for mitoribosome pathology in CLPP-null mice has since been disputed [48,49]. Problems of translation fidelity in mitoribosomes would primarily affect the 13 proteins encoded by mitochondrial DNA to function within respiratory chain complexes [50]. Indeed, it is clear that respiratory complex IV is prominently affected in CLPP-null mice. Although respiratory activity in the brain and muscle appeared almost normal, the complex IV activity in the liver was halved, presumably due to a reduced abundance of the mitochondrially encoded MTCO1 protein (30% in prominently affected testis), despite an *mtCo1* (*Cox1*) mRNA increase in all tissues studied (5-fold in testis) [51]. The biosynthesis of MTCO1 protein is preferentially prone to mitoribosomal stalling, given that its sequence contains one triproline and three diproline motifs in humans via rodents to zebrafish, three diprolines in *Drosophila melanogaster* flies, and two diprolines in *Saccharomyces cerevisiae* yeast [52]. All other mitochondrially encoded proteins have a maximum of two diprolines in their sequence (MTCO3 and MT-ATP8), and a reduced abundance in CLPP-null mice was only observed for MTCO1 and MTND6 in some tissues [51]. Beyond translation impairment, preliminary data also suggested a generalized reduction of respiratory supercomplexes in CLPP-null mouse heart and liver tissues [51]. An altered disassembly and decreased turnover was documented in detail for the respiratory complex I peripheral arm in CLPP-null mice [53]. Thus, assembly/disassembly dynamics may be affected not only for the mitoribosome and the respiratory chain, but also for other protein/RNA complexes as a secondary consequence of CLPP deficiency.

Therefore, a second question of this study focuses on the identification of CLPXP folding/degradation targets to understand the molecular mechanisms of Perrault syndrome. Previous efforts to define the prominent CLPXP degradation substrate proteins in the matrix of bacteria/chloroplasts/mitochondria have yielded widely variable results in different species, and there is an ongoing debate if metabolic enzymes [54,55] or ribonucleoproteins [49] are preferentially targeted. Substrate trap assays with overexpressed mutant CLPP, as well as global proteome profiles in tissues and cells without stress or after stressor administration, and finally databases on the protein interactions of overexpressed or endogenous CLPX have been scrutinized [49], without succeeding to pinpoint an invariably CLPXP-dependent protein. Recent studies of CLPP-null mice and CLPP-mutant patient cells showed the unfoldase CLPX to co-accumulate with selected protein subunits of the mitoribosome and the RNA granule, such as the translation elongation GTPase GFM1, the mitochondrial mRNA processing factor LRPPRC (and also, at least in mice, the G-quadruplex RNA unwinding factor, GRSF1), as well as the CLPX stabilizer POLDIP2 [45,56]. Together with CLPX, accumulation was also shown for the molecular chaperone HSPA9, and the amino acid biosynthesis enzyme OAT, so they were interpreted as CLPP substrate candidates [57,58].

Avoiding the overexpression of exogenous proteins and thus aberrant interactions, two-dimensional blue native gel electrophoresis (BNE) with mass spectrometry (MS) proteomics of consecutive gel slices (dubbed as “complexomics”) was employed in two pioneer studies to define the endogenous physiological interactors of CLP-related AAA+ unfoldases. A complexome study of *E. coli* failed to detect ClpX but showed ClpB to co-migrate with the molecular chaperone DnaK, the translation initiation factor InfB, and the small ribosomal subunit mRNA unfoldase RpsA [59]. A preliminary complexome profile of one CLPP-deficient mouse heart suggested accumulation and disperse migration of CLPX (6-fold) in parallel to the CLPX stabilizer POLDIP2, RNA granule factors GRSF1, TBRG4/FASTKD4 and LRPPRC, translation elongation GTPases GFM1 and GFM2, as well as the AAA+ unfoldase VWA8 [49,53]. It remained unclear whether these observations can be reproduced in more samples and be extended to more severely affected tissues of CLPP-mutant mice or patients. Furthermore, any direct impact of CLPXP on mitoribosomes was not elucidated. While the complete 55S mitoribosome migrates at 2.7 MDa, the 39S mtLSU appears at 1.7 MDa, and the 28S mtSSU at around 1 MDa. The analysis of additional CLPP-null mouse tissues regarding CLPX co-migration and abnormal migration patterns of the endogenous proteins is useful to elucidate the primary events of physiological and non-physiological interactions, as well as CLPX targeting selectivity.

**Figure 1:**
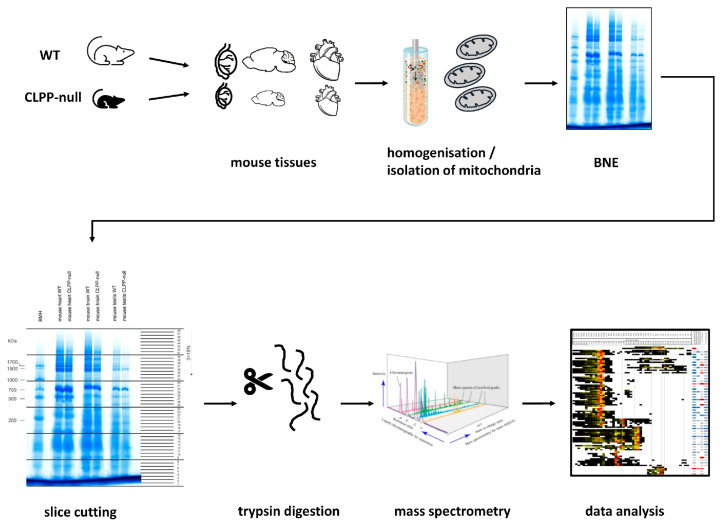
Scheme of experimental flow and data analysis to elucidate CLPP-null triggered anomalies in CLPX interactions with endogenous protein complexes. A higher resolution of the data analysis graph is seen in Supplementary Table S1.

Such complexome profiling efforts were herein performed, using protein extraction with a low concentration of the mild detergent digitonin (so that respiratory supercomplexes and mitoribosomal subunits can remain assembled), followed by BNE and label-free mass spectrometry in 48 sections to quantify the abundance of proteins at each migration position (scheme of the experimental setup in Figure 1). We chose to study CLPP-null testis as maximally affected tissue with the broadest expression profile, the brain as a modestly affected tissue with a broad expression profile, and the heart muscle (despite a lack of clinical affection and its limited expression profile) for the sake of comparison with the previous complexome report. In the present manuscript, the focus was placed on AAA+ unfoldases and RNA processing factors to define the primary events of pathogenesis in PRLTS3. Secondary and compensatory events such as the dysregulation of amino acid metabolism enzymes will be the subject of a subsequent manuscript.

## 2. Results

To obtain complexomics profiles and judge their consistency, mitochondria from three tissues were purified via sucrose gradients before performing protein extraction with a 20% digitonin detergent, blue native electrophoresis (BNE), and label-free quantitative mass spectrometry of 48 gel slices. Subsequent analyses were performed to identify the abundance/accumulations of individual proteins under native conditions, their potential association/co-migration within protein complexes, and their dispersion in mutant samples. The primary data from testis, brain, and heart tissues are provided in Table S1, and were deposited publicly at PRIDE. For validation experiments using quantitative immunoblots, mitochondria were isolated via differential detergent cell fractionation. The crucial findings were then assessed in separate samples via co-immunoprecipitation experiments.

### 2.1. Migration Pattern and Abundance of AAA+ Proteins Indicate That CLPX and VWA8 Both Depend Strongly on CLPP

**Figure 2:**
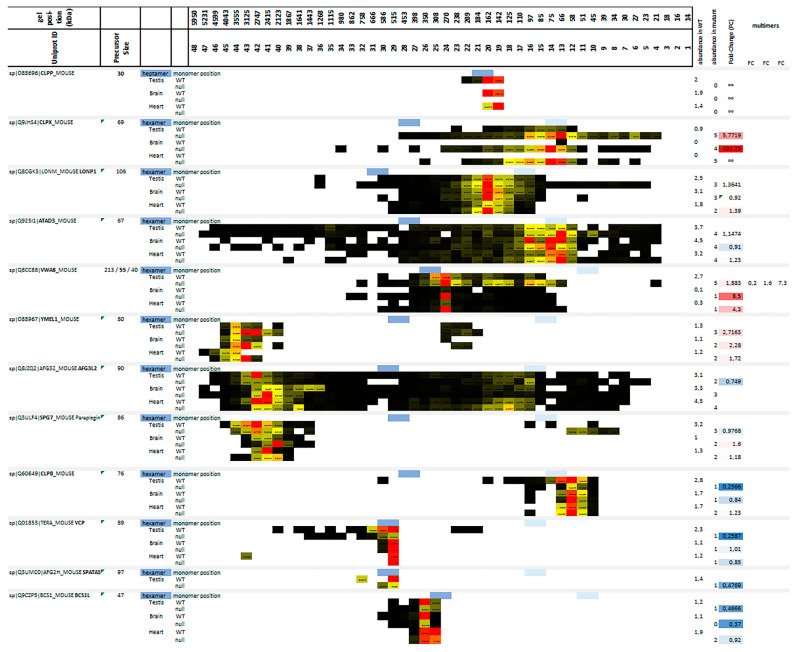
Analysis of mitochondrial AAA+ unfoldases in complexomics profile of WT versus CLPP-null mouse testis, brain, and heart shows selective CLPX and VWA8 accumulation. Successive columns show the identity of each factor providing the UniProt-ID code, then molecular sizes of each precursor protein and potential isoforms according to UniProt database, tissue type, and the genotype for each line. Further columns illustrate the 48 gel slices representing protein complex sizes (from about 6000 kDa on the left to 10 kDa at the right), with white fields representing a lack of detection, while colored fields represent detection with max_norm values (dark brown for lowest, green for medium, bright red for highest abundance). Label-free mass spectrometry was used to quantify abundance in each slice, and the summed abundance across all slices is shown in digits on the right side, displaying mutant total, WT total, and the ratio as fold change (FC) in a heatmap with strongest accumulations in red, while strongest decreases are highlighted in blue. For VWA8, several isoforms are known to exist, and the FC was calculated for three separate migration peaks. Given that AAA+ unfoldases are thought to assemble in homohexamers, the expected migration position for each factor was derived and shown for the monomer (light-blue fields) as well as the homohexamer (dark-blue fields). Confirming the genotypes, CLPP was absent from all mutant tissues. Overall, CLPX, LONP1, and ATAD3 migrated much faster than expected for their homohexameric assemblies, while YMEL1, AFG3L2, and SPG7 migrated much slower than expected for homohexamers. CLPX and VWA8 showed consistent accumulation across the three tissues (LONP1 only in testis and heart) and migrated in a more disperse pattern when CLPP was absent. In CLPP-null more than WT tissues, CLPP and VWA8 migration positions overlapped. A higher resolution of the data analysis graph is seen in Supplementary Table S1.

As expected, CLPP protein was absent from mutant samples and migrated in wildtype (WT) samples at positions that correspond to a homoheptameric ring (Figure 2). However, no CLPP peptides were detected where the CLPXP proteolytic barrel would run in BNE (expected molecular size around 565 kDa), and only 5% of CLPX co-migrated with the homoheptameric CLPP ring in the WT testis. Also, unexpectedly, the abundance of endogenous CLPX, LONP1, and ATAD3 in the WT tissues peaked at the monomer to dimer positions, with no CLPX at all being detected at the expected homohexamer position. These surprising multimerization deficits may be explained by two alternative scenarios: (1) Our samples were frozen and treated with sucrose and digitonin, possibly resulting in the dissolution of relatively unstable complexes. However, blue native gels with samples that were never frozen also showed anti-CLPP immunoreactivity at the homoheptamer migration position, rather than the expected CLPXP proteolytic barrel. The proteolytic CLXP chamber appears only when CLPP cannot complete its function (see [45], Supplementary Figure EV3 B). (2) Alternatively, most CLPX may serve unfoldase–foldase–chaperone functions independently from CLPP degradation activity, for example, assisting PLP to access the appropriate fold within ALAS for porphyrin biosynthesis. While we are studying endogenous mitochondrial proteins in the absence of degradation stress during slow growth, the previously published structural analysis of CLPXP barrels employed overexpressed and purified bacterial proteins, mixed at saturating conditions, from cells growing at logarithmic rates where the protein degradation capacity may be maximized [60].

The observation that mature CLPX accumulated and migrated not only at its expected monomeric position around 60 kDa, but physiologically up to sizes around 250 kDa, and in CLPP-null tissues until sizes of 450 kDa, made it possible to use co-accumulation, co-migration, and dispersion patterns as criteria to identify potential CLPX interactors in the matrix. Overall, the scarcity of mitochondrial matrix proteins that co-migrate with CLPX, as well as showing accumulated abundance upon CLPP deficiency, was very helpful to identify candidate interactions, which can be validated using further technical approaches.

Several migration positions existed for VWA8, and they were roughly compatible with dimer, trimer, and homohexamer sizes for the medium mouse isoform around 55 kDa, but not for the small (40 kDa) and large (213 kDa) isoforms (Figure 2). The inner membrane-associated AAA+ proteases YME1L1, AFG3L2, and SPG7 showed unexpectedly high migration sizes, co-migrating around 3 MDa, consistent with reports that YME1L1 exists in a complex with PARL and SLP2 [61], while AFG3L2 and SPG7 are tethered to PHB1 [62] (Figure 2). Migration positions compatible with the expected homohexameric assembly were observed only for CLPB, VCP, SPATA5, and BCS1L.

CLPP deficiency caused accumulation and dispersed migration in overlapping positions for the matrix unfoldases CLPX and VWA8 in all three tissues (Figure 2), the matrix unfoldase/protease LONP1 accumulation and dispersion in two tissues, and inner membrane unfoldase YME1L1 accumulation at much higher migration positions than CLPX (this metalloprotease acts on respiratory complex IV [63] and in the mitochondrial intermembrane space, rather than the matrix), while the other mitochondrial AAA+ unfoldases appeared unchanged. Thus, only VWA8 shows similarity to CLPX in mutant tissue. Importantly, a meta-analysis of proteins that pulled VWA8 as prey in interaction assays according to the BIOGRID database (accessed on 7 November 2022) identified the mitochondrial RNA granule and mitoribosomal factors, as well as the respiratory chain complexes, as putative targets of VWA8 (Figure S1), in good overlap with previously reported CLPX targets [45,49,53,56].

### 2.2. Co-Migration of mtSSU Monomeric Factors with ERAL1 as Expected, but Only mtLSU Intermediate Assemblies Co-Migrate with CLPX and VWA8

**Figure 3:**
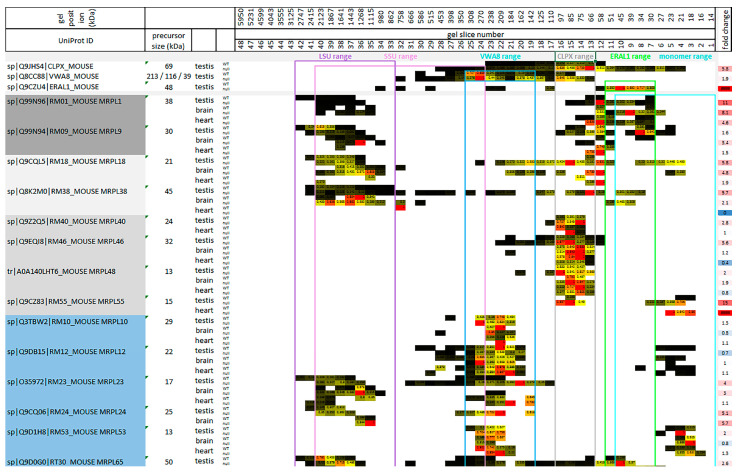
mtLSU factors in complexomics profile of WT versus CLPP-null mouse testis, brain, and heart. To define possible interactions, migration patterns were compared with the detection range of AAA+ unfoldases CLPX (grey box) and VWA8 (dark-blue box), as well as the mtSSU-folding rRNA chaperone ERAL1 (green box). For orientation, the range of monomeric mitoribosomal factors (light-blue box), the assembled mtLSU (purple box), and the assembled mtSSU (lavender box) is shown. All detectable mtLSU and mtSSU complexomics data were compiled in Table S1. The format is analogous to Figure 2. Numbers in the right margin represent the null/WT total abundance ratio as fold change (FC). The heatmap illustrates the strongest accumulations in red, while the strongest decreases are highlighted in blue. Overall, CLPX and VWA8 did not co-migrate with fully assembled LSU or monomeric proteins, but instead with two different intermediate assemblies of the LSU. The ERAL1 detection range showed almost no overlap with the CLPX range in WT tissues. A higher resolution of the data analysis graph is seen in Supplementary Table S1.

To understand mechanistically how CLPXP targets the mitoribosomal translation machinery, the physiological and pathological migration range of target-selecting CLPX was compared firstly to mitoribosomal proteins (Figure 3), then to other ribonucleoproteins (Figure S2), furthermore to PLP-associated proteins and additional chaperones (Figure S3), and finally to the mitoribosomally translated respiratory chain components (Figure S4).

Given a previous claim that the rRNA chaperone ERAL1 mediates the CLPP-null effect on mitoribosomes [45], it was surprising to find that the CLPX migration range in BNE gels had little overlap with the positions where ERAL1 was detectable (Figure 3). In agreement with previous reports that ERAL1 acts in the assembly of the mtSSU with its 12S rRNA [46,47], ERAL1 migrated around 50 kDa in overlap with some mtSSU factors at monomeric positions (Figure 3 and Table S1).

**Figure 4:**
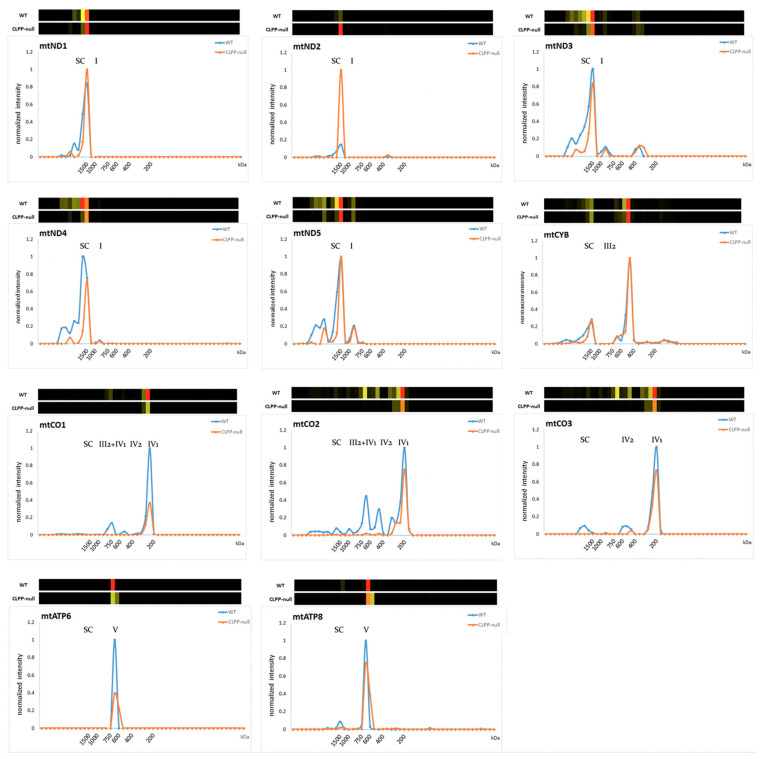
Migration profiles of mitochondrially translated factors in testis as most severely affected tissue. Each subpanel shows the amount of a protein at different migration positions, above illustrating it as heat map, and below providing a plot of relative abundance on the *Y*-axis, with the *X*-axis representing the 48 slices of BNE gels with the scale of approximate molecular mass in kDa. The expected size of fully assembled monomeric complexes is denoted in each panel as Roman numeral, with Arabic numerals behind indicating homo-multimerization status (1 for monomer, 2 for dimer). Monomeric CI runs around 1 MDa, dimeric CIII around 0.5 MDa, monomeric CIV around 0.2 MDa, CV around 0.7 MDa, and supercomplexes 0–4 (SCs) in the range around 1.5 MDa and above [67,101,102]. Although not distinguished in the panels for space reasons, CI+CIV are known to associate, before supercomplex S0 containing I + III2, and supercomplex S1 containing I + III2 + IV are assembled [102]. The multimerization of CIV1 to CIV2 at about 0.45 MDa and its association as CIII2-CIV1 at about 0.7 MDa are known higher assemblies of MTCO1-2-3 [103]. The defect of homo-multimerization and association with other respiratory complexes was particularly severe for complex IV, as seen for proteins MTCO1-MTCO2-MTCO3.

In contrast, CLPX migration occurred at higher molecular weights than monomeric proteins and lower than completely assembled mtSSU/mtLSU complexes. This finding casts doubt on the proposed CLPXP role for functional mitoribosomes that were stalled during translation. Instead, CLPX co-migrated with intermediate assemblies of specific mtLSU factors (see Figure 3 and the Graphical Abstract in Section 3). Such mtLSU intermediate assemblies have been observed in BNE gels before and represent functional clusters [64,65,66,67,68,69], so they do not seem to be artifacts of tissue freezing. Beyond their positions within the CLPX range, these mtLSU factors were also conspicuous for their relatively strong accumulation and their dispersed migration in CLPP-null tissue, and therefore are credible CLPX targets. They include the stalk factors MRPL1 (average accumulation: 8-fold) and MRPL9 (2-fold), as well as a previously described [65,66,67] mitochondria-specific cluster among central protuberance components, namely MRPL40 (2-fold), MRPL46 (2-fold), MRPL48 (2-fold), and MRPL55 (also known as bL31m; 15-fold accumulation in testis, infinite in heart), presumably together with MRPL18 (4-fold) and MRPL38 (3-fold) (see Figure 3 in [70], Figure 4 and extended Figure 8 in [71], and [69]). The L1 stalk of the mtLSU has been implicated in tRNA translocation [72], while the L18-L38-L40-L46-L48 cluster serves to hold the mitochondrial tRNA-valine or tRNA-phenylalanine in the central protuberance (see Figure 3 in [70,73]). It is interesting to note that MRPL18, MRPL25, MRPL46, MRPL48, and MRPL55 are not phylogenetically conserved in *Saccharomyces cerevisiae,* just like CLPP. Three proteins showed much higher migration positions in the CLPP-null tissues, namely MRPL18, MRPL38, and MRPL46, suggesting that tRNA-valine integration problems persist during ever larger assembly stages, and show a very similar dispersion/accumulation pattern in complexomics to CLPX. Given that CLPP loss triggers the identical phenotype in humans as mutations in the mitochondrial tRNA ligases HARS2 and LARS2, a common denominator of Perrault pathogenesis could be tRNA processing. Our observations may highlight an impact of CLPXP on the folding and assembly of tRNA^Val/Phe^ with mtLSU central protuberance (CP). In contrast to the quite selective CLPP-null effect on only a few mtLSU factors, non-selective accumulation was observed for practically all mtSSU factors, without CLPX co-migration (Table S1), possibly due to mtSSU interaction with accumulated LRPPRC/SLIRP/HARS2/TARS2 plus tRNAs.

The VWA8 migration peak was around 200 kDa at higher molecular weights than the 80 kDa CLPX peak, while the two migration ranges overlapped. Again, VWA8 co-migrated with an mtLSU intermediate assembly complex, suggesting that it acts during the association or dissociation stage rather than the repair of complete mitoribosomes. This is consistent with previous observations that the yeast VWA8 ortholog Midasin/Rea1 acts in the maturation/assembly of pre-mtLSU central protuberance and the L1 stalk with 5S rRNA [74]. In mouse, VWA8 co-migration concerned, on the one hand, the MPRL10-MRPL12-MRPL53 proteins as components of the LSU L7/L12 stalk (a subcomplex that was already reported and co-migrates also with LRPPRC and SLIRP [64,65]), but the CLPP-null mutation did not cause them to accumulate or migrate differently. The L7/L12 stalk is the docking site of translation elongation GTPases (orthologs of GFM1/2) [75]. On the other hand, the VWA8 co-migration with the LSU NPET (nascent polypeptide exit tunnel) wall proteins MRPL23-MRPL24 [70] was accompanied by altered migration and accumulation (on average, 3-fold for MRPL23, 5-fold for MRPL24). MRPL65 was previously named MRPS30, and its position in the latest mtLSU assembly is shown in [70] Figure 1 (right panel). MRPL65 also showed a VWA8 co-migration that appeared altered upon CLPP deficiency, as well as accumulation in the mutant (3-fold). Thus, when mitoribosomal translation fidelity is impaired in CLPP-null tissues due to CLPX-dependent folding problems at the mtLSU central protuberance, the unfoldase function of VWA8 might target specific NPET components in mtLSU disassembly intermediates.

Serendipitously, we observed the distribution of MRPS36/KGD4 to be well above the size of the completely assembled mitoribosome, arguing against its integral role for the mtSSU and supporting recent evidence that it is a subunit of the mitochondrial alpha-ketoglutarate dehydrogenase complex [76,77].

All other ribonucleoproteins were assessed, and 16 candidates were conspicuous in all three tissues (Figure S2), with translation factors occupying a prominent place. Among the disease proteins responsible for Perrault syndrome, HARS2 was prominent because of its strong accumulation in CLPP-null tissue, as previously reported upon quantitative immunoblots [17], and here, the complexomics profile revealed a parallel distribution of CLPX and HARS2 from their monomeric sizes (precursors of 69 and 23 kDa, respectively) up to molecular sizes around 250 kDa. Similarly, a close co-migration with CLPX was documented for GFM1 from its monomeric size (84 kDa) to positions around 250 kDa, in addition to very strong accumulation (average 10-fold). A recent review of putative CLPXP substrates in different species concluded that the translation elongation GTPase GFM1/GFM2 orthologs are exceptionally consistent candidates [49]. Curiously, the extra-mitochondrial translation initiation complex core factor EIF3C (precursor size 106 kDa) also appeared greatly accumulated in CLPP-null mitochondria (average 5-fold) and showed co-migration across the entire CLPX distribution range. EIF3C is a core factor in the EIF3 complex that recruits tRNA-methionine during pre-initiation to scan for start codons versus internal ribosomal entry sites under stress conditions [78,79,80,81].

In addition, several proteins with localization at the mitochondrial RNA granule co-migrated with CLPX. Moderate accumulation in CLPP-null tissue (LRPPRC: 3-fold, SLIRP: 12-fold), but clear dispersion, was observed for both components of the LRPPRC-SLIRP complex, which associates preferentially with the 5′-untranslated region of *mtCo1* and *mtCo3* mRNA, delivering them to the mRNA channel in the mtSSU via PTCD3/MRPS39 [82], and is crucial for isolated complex IV deficiency [83,84,85,86]. While they showed only minor overlap with the physiological CLPX migration range, their migration position coincided with the high-molecular-weight positions reached by accumulated CLPX in the mutant and coincided with the VWA8 migration range. Much stronger accumulation (ratios up to infinite values) and CLPX/VWA8 co-migration/dispersion were documented for additional RNA-binding PPR-domain-containing factors such as PTCD1 (and PTCD2) as pseudouridylation enzymes, which processes rRNA before integration into the mtLSU [87], but also performs the processing of tRNA-ligase for leucine (LARS2) and controls respiratory complex IV activity [88,89]. Given that mutations in the mitochondrial leucine tRNA-ligase LARS2 cause the Perrault syndrome just like CLPP deficiency [90], this observation may further strengthen the notion that RNA processing is important in this pathogenesis. A similar massive upregulation and CLPX co-migration/dispersion were also apparent for the G-quadruplex RNA unwinding factor GRSF1, which mediates protective effects against iron overload and ferroptosis [91], and which interacts with the mitochondrial RNase P complex subunit TRMT10C/MRPP1 [92]. Modest accumulations were also found for PLP-dependent SHMT2 and MTHFD2, which are needed for tRNA methylation in mitochondria [93,94], otherwise stalled mitoribosomes would occur [95]. Jointly, these observations suggest that specific mitoribosomal translation factors may be the main physiological targets of CLPXP, but particularly upon CLPP deficiency, the excess CLPX/VWA8 may also target RNA granule components.

Accumulation, CLPX/VWA8 co-migration, and dispersion in CLPP-null tissues were also documented for various molecular chaperones, and enzymes whose activity is modulated by the chaperone PLP (Figure S3). Importantly, the Pyridoxal Phosphate Binding Protein (PLPBP or PROSC) showed migration positions up to 70 kDa in mutant brain tissue instead of its WT position around 30–35 kDa. As expected, as a consequence of the gain-of-function of CLPX and PLP, their established target ALAS2 as a rate-limiting enzyme of porphyrin/heme biosynthesis in most tissues showed massive accumulation. Strong accumulation, dispersion, and co-migration with CLPX were also apparent for the PLP-dependent enzyme OAT, and similar accumulation also concerned the enzyme ALDH18A1, as previously reported in global proteome profiles [56,96]. Both factors have connected pathway functions in the interconversion of amino acids such as proline, glutamate, and arginine. Smaller accumulations and some dispersions were observed for the pyruvate homeostasis enzymes PDK1, PDK3, and PDPR.

In view of the phylogenetically conserved interaction between CLPX and HSP70 family members in the chaperone pathway, it was no surprise to find accumulations of HSPA9 (Mortalin) and the mitochondrial HSP70 co-chaperones/modulators GRPEL1-GRPEL2-DNLZ-CCDC127 [97,98,99], but also TRAP1 as a mitochondrial member of the HSP90 family showed similar accumulation. However, both chaperones have molecular sizes that are very similar to the CLPX precursor around 69 kDa, so their co-migration would be a coincidence. Overall, the loss of CLPP peptidase and the accumulation of CLPX unfoldase has a profound impact on factors that interact with the chaperone PLP and determine folding/assembly.

### 2.3. CLPP-Null OXPHOS Complexes Affected More by Assembly Than Translation Problems

The CLPXP complex has a prominent impact on mitoribosomal translation, which is responsible for the biosynthesis of 13 well-characterized proteins that have key roles in the respiratory chain complexes CI, CIII, CIV, and CV. While these factors are embedded in the mitochondrial inner membrane, many regulatory factors, assembly factors, and factors that assist in the flow of electrons are assembled around them in complexes that protrude in the matrix space. This matrix immersion is particularly true for the N-module of CI, and indeed, the three core proteins of the N-module (NDUFS1, NDUFV1, NUDFV2) were shown to be CLPP-dependent in their exceptionally high turnover rate [53]. Analyzing the complexome profiles regarding these N-module proteins (Figure S4 top), they were found to co-migrate with CLPX across a wide range in WT samples, and even more in CLPP-null tissues. Interestingly, the abundance of NDUFS1-NDUFV1-NDUFV2 was increased selectively in CLPP-null slices, where they co-migrated with CLPX (Figure S4, top, fold-changes in right margin), suggesting inefficiency and slowed turnover during assembly interactions. In contrast, their abundance was decreased in the slices that represent a fully assembled complex I, suggesting that their integration into their N-module position is impaired. Altogether, these complexome data confirm a role of CLPXP for the CI N-module core factor, but apparently regarding their assembly/disassembly rates rather than their degradation.

The complexomics profile of mitochondrially encoded/translated factors in the respiratory chain again showed a lowered abundance rather than accumulation in CLPP-null tissue, in good agreement with the notion of impaired mitoribosomal translation fidelity. Prominently strong reductions were found in testis, as the most severely affected tissue of CLPP-null mice that model the complete infertility of PRLTS3 patients. The reductions reached levels of 0.36-fold for MTND4, 0.32-fold for MTCO1 (COX1), and 0.36-fold for MTCO2 (COX2) (Figure S4, middle, right margin).

However, deficits of assembly seemed much more severe than deficits of translation. As shown in Figure 4, monomeric complexes I-III-IV-V formed quite adequately in CLPP-null testis as the most severely affected tissue, but the multimerization steps to form supercomplexes (SCs) were generally deficient. An almost absent integration into higher-order SCs was observed for MTND3-MTND4-MTND5, and for MTCO1-MTCO2-MTCO3, practically no assembly into the CIV2 dimer, the CIII2-CIV3 intermediate assembly, or SCs 0–4 was detected (Figure 4) (mutant testis showing fold changes of 0, 0.04, and 0, respectively, in the migration range from IV2 to SC). It is therefore interesting to note that in WT tissue, VWA8 co-migrated with the CI Q-module assembly factor NDUFAF3 and the ND1 module assembly factor TIMMDC1, as well as the mitochondrial CI intermediate assembly (MCIA) complex members TMEM186, NDUFAF1, ECSIT, ACAD9, and TMEM126B (which are responsible of the ND2 module), all of which accumulated in CLPP-null testis (Table S1). An uncommonly strong 17-fold accumulation, together with a migration shift to a much higher, possibly dimeric/trimeric position, was noted in CLPP-null brain for SFXN4, which was recently identified to modulate iron homeostasis and act as an MCIA component for CI assembly [100]. Accumulation (2.7-fold) was also documented for SLC25A28 as a mitochondrial iron transporter upon CLPP depletion, but this was detectable only in testis tissue.

Furthermore, co-migration with CLPX in WT tissue was observed for practically all components of respiratory CIV (Figure S4). As a CIV component protein, which showed outstanding accumulation in CLPP-null tissues (8-fold in testis) and co-migrated with CLPX in its most abundant positions (slices 12–16), the complexome profiles identified COX15 (Figure S4, bottom). This protein is not a structural constituent of the fully assembled complex IV but is essential for its biogenesis and assembly. Curiously, COX15 is not immersed in the matrix, but has five transmembrane domains that localize it as homo-oligomer in the mitochondrial inner membrane [104,105]. Thus, it is unlikely to be a direct target of CLPXP functions, but its selective strong affection might be indirect: CLPP-null mice are known to have elevated hemoglobin [106] and probably also heme levels, and COX15 uses two heme-O molecules to generate heme-A molecules that are needed as cofactors to mediate CIV assembly and activity [104,107].

Overall, the impact of CLPXP on the respiratory chain appears strongest on CIV and CI, causing a deficit in total levels rather than the accumulation seen for mitoribosomal translation factors, but respiratory chain SC assembly problems were stronger than peptide chain synthesis problems, and may be secondary to the iron/heme homeostasis alteration via SFXN4-MCIA for CI, and via COX15 for CIV.

### 2.4. Quantification of Heavy Metals in CLPP-Null Tissue Demonstrates Significant Increases in Iron, Molybdenum, Cobalt, and Manganese

In view of the known role of excess CLPX for ALAS overactivity for heme biosynthesis, and of the accumulation of iron-associated SFXN4-COX15 as a potential explanation for respiratory complex assembly deficits, the question arose if iron and other heavy metals as well as heme are dysregulated and enhance PRLTS3 pathology in nervous circuits. The deposition of excess iron was documented in many neurodegenerative disorders [108]. Postmitotic neurons in brain tissue may be particularly vulnerable to the chronic impact of heavy metal toxicity via reactive oxygen species, ferroptosis, and inflammation. The iron analysis was extended to a survey of other heavy metals (see Table S2), since excess protoporphyrin is toxic and may also chelate magnesium, zinc, copper, nickel, vanadium, cobalt, and manganese [109,110,111,112,113,114].

As summarized in Table 1, 40–20% increases were observed for iron, molybdenum, cobalt, and manganese. These data are consistent with the overactivity of heme production by ALAS and indicate a more widespread metal pathology. The tissue excess of molybdenum and cobalt suggests increased levels of the pterin-based molybdenum cofactor (Moco) [115] and of corrin-ring-based cobalamin (vitamin B12), respectively, while the excess of manganese is compatible with maximal activity of Mn-containing enzymes such as arginase or mitochondrial superoxide dismutase. Neurotoxicity is well established for manganese overdosage [116], while molybdenum toxicity represses growth and fertility [117]. Overall, the elevation of iron and several other heavy metals in CLPP-null tissue may contribute to the impaired homeostasis of heme and downstream heme-dependent assemblies of respiratory CI and CIV.

### 2.5. Validated Accumulation of VWA8, CLPX, PLPBP, GFM1, MRPL18, MRPL38, HSPA9, TRAP1, COX15, PTCD1, ALDH18A1, and OAT in Quantitative Immunoblots

**Figure 5:**
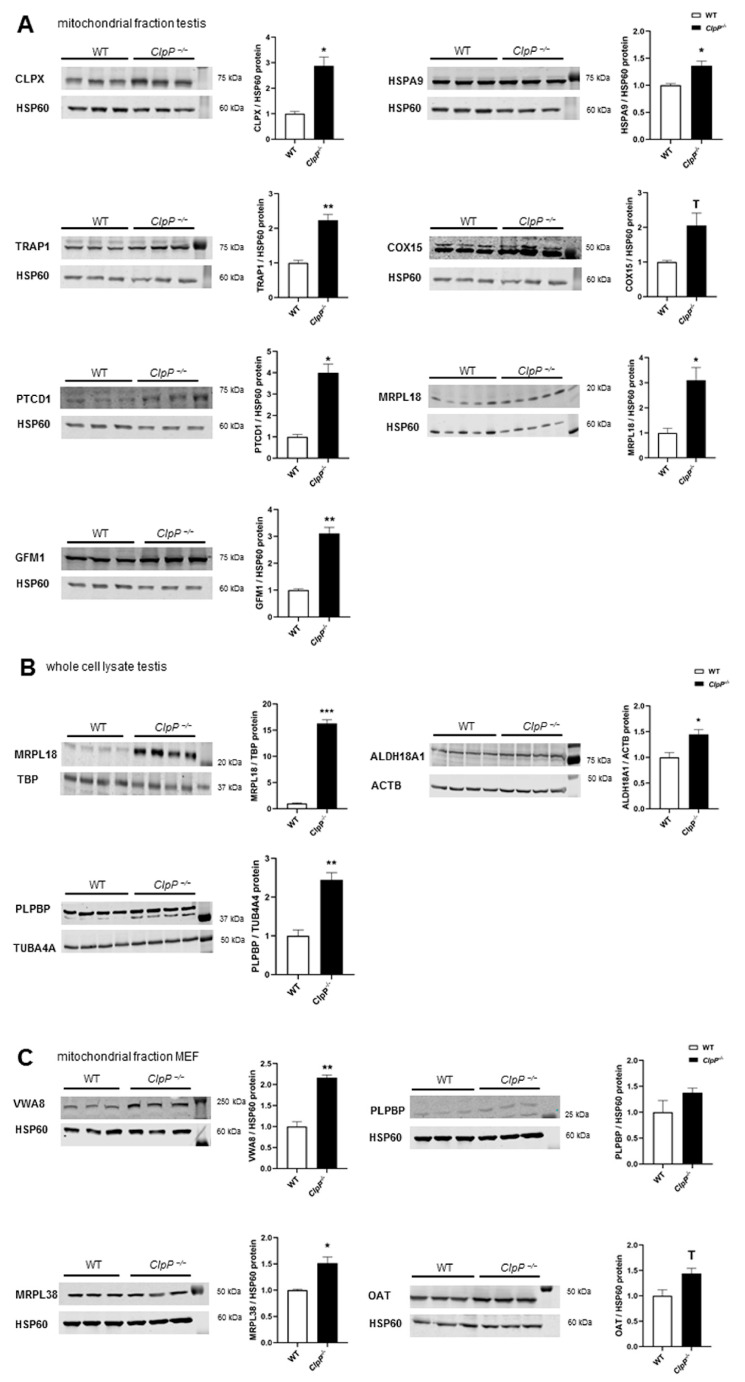
Experiments to assess mass-spectrometry abundance data using quantitative immunoblots (**A**) Quantitative immunoblots in P21 testis subjected to differential detergent fractionation using the mitochondrial protein HSP60 as loading control to normalize abundance. (**B**) Quantitative immunoblots in 5-month-old testis tissue, whole cell lysates. Protein abundance levels were normalized against TBP, ACTB, or TUB4A4 levels. (**C**) Quantitative immunoblots in MEF cells, subjected to differential detergent fractionation with mitochondrial HSP60 as normalizing protein. * *p* < 0.05; ** *p* < 0.01; *** *p* < 0.001; T (statistical trend) 0.05 < *p* < 0.1.

While the complexome profiles above assessed the consistency between different tissues for all abundant mitochondrial proteins, subsequent validation experiments using quantitative immunoblots focused on relevant factors and tested their anomalies statistically, using several samples from testis or murine embryonic fibroblasts (MEFs) depending on variant abundances and cross-reacting bands of both tissue expression patterns. For these experiments, mitochondrial fractions were isolated using differential detergents from cytosolic and nuclear fractions, and a specificity and sensitivity analysis was performed for antibodies that were later used in co-immunoprecipitation studies. A convincing detection of endogenous protein levels (see Figure 5A) was achieved for CLPX and VWA8 as AAA+ unfoldases, PLPBP as a PLP-chaperone homeostasis protein, HSPA9 and TRAP1 as molecular chaperones, COX15 as a heme-A synthase for CIV assembly, PTCD1 as an RNA granule component, MRPL38 and MRPL18 as mtLSU members, GFM1 as a translation elongation factor, and OAT as a PLP-dependent amino acid homeostasis enzyme.

In mitochondrial fractions from testes at postnatal day 21 (P21), where spermatogenesis has started but apoptotic cell loss is not yet noticed in mutants, using HSP60 as a fraction marker and loading normalizer, accumulations were confirmed for CLPX (2.9-fold, *p* = 0.003), HSPA9 (1.4-fold, *p* = 0.04), TRAP1 (2.2-fold, *p* = 0.01), COX15 (2.1-fold, *p* = 0.10), PTCD1 (4.0-fold, *p* = 0.01), MRPL18 (3.1-fold, *p* = 0.02), and GFM1 (3.1-fold, *p* = 0.01). Solubilizing more protein via the SDS detergent in RIPA buffer, lysates of total testis tissue showed an even stronger accumulation of MRPL18 (16.3-fold; *p* = 0.0002) and also detected significant accumulations of ALDH18A1 (1.4-fold; *p* = 0.02) and of PLPBP (2.45-fold; *p* = 0.001). In mitochondrial fractions of MEFs, accumulations were replicated for VWA8 (2.16-fold, *p* = 0.003), MRPL38 (1.5-fold, *p* = 0.04), and OAT (1.4-fold, *p* = 0.05), but without significance for PLPBP (1.4-fold, *p* = 0.23) in these cells, in contrast to the severely affected CLPP-null testis. Overall, the quantitative immunoblots validated the principal complexome findings.

### 2.6. RT-qPCR Analysis of Transcriptional Regulations

**Figure 6:**
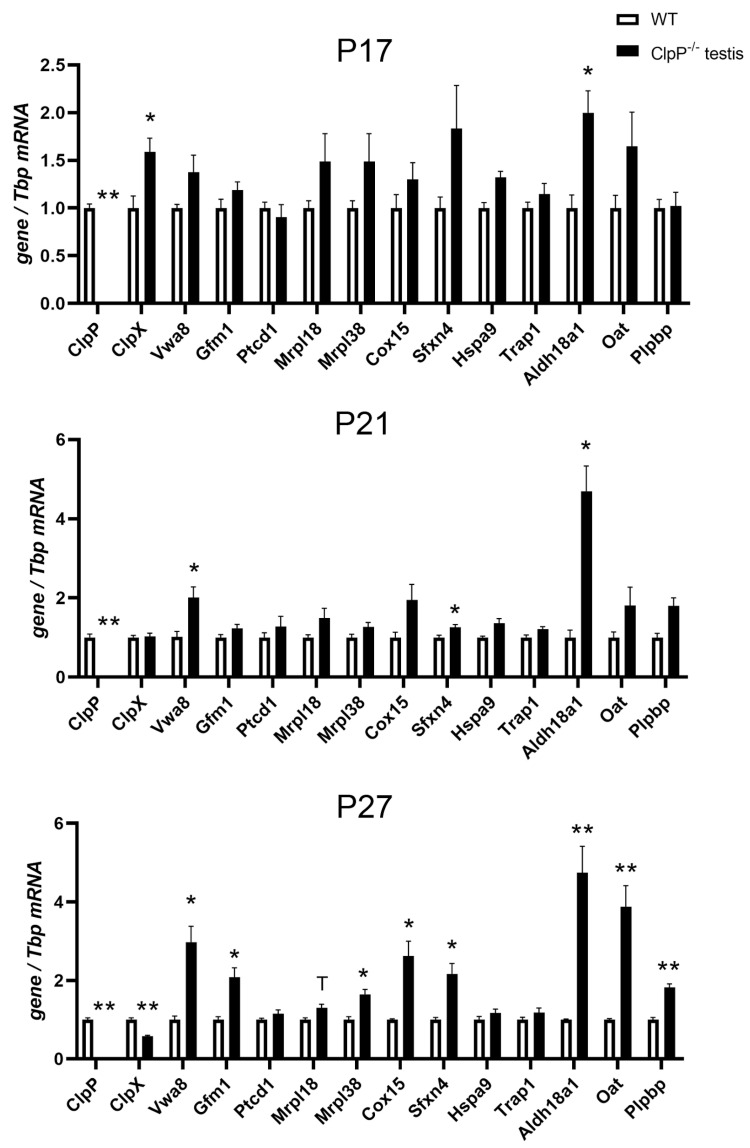
RT-qPCR studies in P21 and P27 testis used *Tbp* mRNA levels as loading controls to normalize expression. * *p* < 0.05; ** *p* < 0.01; T (statistical trend) 0.05 < *p* < 0.1.

To understand if protein accumulations are due to delayed turnover as a consequence of CLPXP proteolysis deficits, or due to transcriptional upregulation as a possible compensatory effort, the expression of individual key factors was quantified via a reverse transcriptase quantitative polymerase chain reaction (RT-qPCR). Expression upregulations of *Hars2* and *mtCo1* in testes have been reported previously [51]. To now elucidate the sequence of pathogenesis events, testis tissue was studied at postnatal days P17 and P21 (pre-manifestation) as well as P27 (post-manifestation). At the P27 stage, many transcripts show dysregulated levels, and it has to be taken into account that selective cell losses may account for some of the changes due to the rapid apoptosis of spermatids at the end of meiosis-I. As shown in Figure 6, *Clpp* transcripts were absent in all CLPP-null samples, as expected, and *Clpx* transcripts were downregulated at P27, presumably as a cellular effort to compensate the pathological CLPX protein accumulation. Conversely, however, *Vwa8* transcripts were upregulated, suggesting that VWA8 protein accumulation serves to achieve some folding/assembly tasks that were inadequately performed by mutant CLPXP. Importantly, significant upregulations of *Aldh18a1* mRNA were observed already at P17, P21, and P27 (2.0-fold at *p* = 0.03, 4.7-fold at *p* = 0.02, 6.1-fold at *p* = 0.002, respectively). Also, the *Oat* transcripts seemed consistently elevated at P17 (1.65x, *p* = 0.21), P21 (1.81x, *p* = 0.22), and P27 (3.48x, *p* = 0.002), but this became significant later than for *Aldh18a1*.

These findings suggest that, from the pre-manifest stages, cells maximize the metabolic capacity of these enzymes to counteract their insufficient activity or/and impaired folding/assembly. At later, post-manifest P27 in testes, similar transcript upregulations were also observed for translation factors (*Gfm1*, *Mrpl38*) and respiratory complex assembly factors (*Cox15*). Interestingly, transcripts of the iron-binding CI assembly factor *Sfxn4* seemed to have already increased at P17 (1.84×, *p* = 0.20), with significantly upregulated mRNA levels at P21 (1.26×, *p* = 0.03) and P27 (2.17×, *p* = 0.04). The transcript of the PLP-binding protein *Plpbp* was also elevated at P27 (1.8×, *p* = 0.003), reflecting the complexome protein abundance results.

### 2.7. Co-Immunoprecipitation of CLPX Confirms Interaction with MRPL18, GFM1, and OAT

To validate associations of CLPX with target proteins that were suggested by complexomics data, the co-immunoprecipitation (CoIP) of CLPX and the detection of endogenous protein complexes in tissue would be optimal. However, this approach depends firstly on the sufficient abundance of target proteins, secondly on the high quality of antibodies for precipitation and detection, and thirdly on finding appropriate detergent/salt conditions. The CoIP conditions must disrupt the membranes of cells/mitochondria and dissociate mitoribosomes or respiratory chains sufficiently to permit the purification of interaction partners on beads, but these conditions must not interfere with the stable binding between CLPX and its substrate. We concentrated the validation work on GFM1 as a translation elongation GTPase, the MRPL18/MRPL38 cluster as mtLSU-CP components that are folded around tRNA^Val/Phe^, and the amino acid homeostasis enzyme OAT, which is soluble in the mitochondrial matrix. All of them were reported as targets of PLP binding [118,119,120,121]. The abundance of all these endogenous proteins is usually too low for detection in CoIP experiments, barely reaching the detection threshold in the testis and liver, where levels are maximal according to The Human Protein Atlas webserver (https://www.proteinatlas.org/ENSG00000166855-CLPX/tissue, last accessed on 12 December 2022). As expected, however, the specific CLPX-MRPL38-GFM1-OAT immunoblot signals became reproducibly visible in CLPP-null tissue.

**Figure 7:**
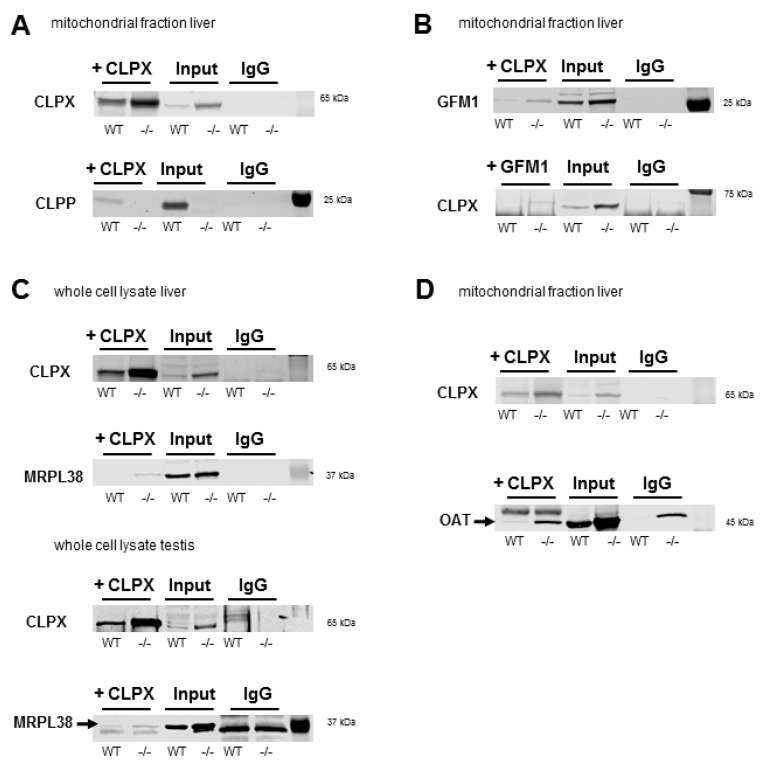
Experiments to validate mass-spectrometry complexomics via co-immunoprecipitation. The precipitation target protein is highlighted with the + sign. (**A**) Validation of IP by pulling with CLPX antibody from Invitrogen, detecting CLPX with antibody from Abcam. Validation of CLPX-co-immunoprecipitation using CLPP (anti-CLPP antibody from Proteintech) as positive control of interaction and assessing correct genotyping of CLPP-null tissues. (**B**) CoIP analyses in liver mitochondrial fractions used anti-CLPX (Invitrogen) and anti-GFM1 (Proteintech) antibodies with sufficient sensitivity and specificity to detect endogenous proteins, pulling down each of the two interaction partners as bait to assess the association with its prey. When the endogenous abundance of these proteins was at the threshold of detection in WT cells, their pathological accumulation in CLPP-null tissues made it possible to demonstrate associations. (**C**) CoIP analyses in whole-cell liver and testis lysates, pulling with anti-CLPX (NSJ Bioreagents, San Diego, CA, USA) and detection of CLPX (Abcam) as control also gave specific bands for MRPL38 (Invitrogen) in both tissues. In testis tissue, a slightly lower unspecific band appeared in the IgG control samples; the height of the correct band is marked with an arrow. (**D**) CoIP analyses in liver mitochondrial fractions pulled with anti-CLPX (Invitrogen) and confirming the IP with anti-CLPX (Abcam) showed specific bands for OAT (Invitrogen). A slightly higher unspecific band was detected in CLPP-null IgG control sample; the correct height of the band is marked with an arrow.

As shown in Figure 7, we prioritized GFM1, which was the most consistent CLPXP target among all surveys in various organisms [49]. Anti-CLPX antibodies (Invitrogen, Karlsruhe, Germany) co-immunoprecipitated GFM1 (detection by anti-GFM1 from Proteintech, Planegg, Germany) from liver mitochondrial fractions. Conversely, upon the CoIP of GFM1 (Proteintech) a CLPX band (detection by anti-CLPX from Abcam, Cambridge, UK) of expected size was detectable in CLPP-null liver whole cell lysates.

Given that the antibody against MRPL18 was of insufficient specificity for CoIP, MRPL38, as its closest interaction partner with tRNA-valine, was analyzed. Again, the CoIP of CLPX (anti-mouse-CLPX from NSJ, San Diego, CA, USA) was able to pull MRPL38 in CLPP-null liver whole cell lysates more than WT (detection by Invitrogen anti-MRPL38), as well as in WT and mutant testis whole-cell lysate (detection by Invitrogen anti-MRPL38), as demonstrated by the appearance of a specific band with the correct size and stronger abundance in the CLPP-null sample. Furthermore, the CoIP of CLPX (Invitrogen) was also able to pull OAT (detection by Invitrogen anti-OAT), again from a CLPP-null liver mitochondrial fraction. While the antibodies against MRPL38 and OAT were specific enough for immunoblot detections, they did not have the quality to be used in the converse CoIP of MRPL38 and OAT to detect the associated CLPX.

Although the CoIP data on WT tissues could not always unequivocally demonstrate physiological interactions between the two endogenous proteins, our CoIP findings in the pathological CLPP-null tissues coincide with previously published overexpression experiments and may underlie PRLTS pathology. Overall, these findings provide evidence that CLPX modulates the access of PLP not only to ALAS (thus modifying the folding/activity/stability of this target protein), but similarly also interacts with GFM1/mtLSU-CP proteins/OAT as known targets of PLP.

## 3. Discussion

**Figure 8:**
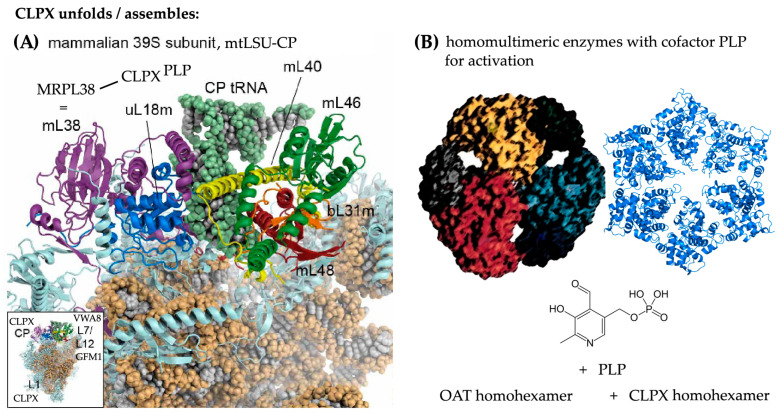
Schematic overview of endogenous CLPX (and VWA8) AAA+ unfoldase targets in the mitochondrial matrix. The CLPP-null complexome profiling data in Table S1 provides evidence that (**A**) a mitoribosomal LSU intermediate assembly composed of MRPL18, MRPL38, MRPL40, MRPL46, and MRPL48 co-migrates and co-accumulates with CLPX, and that (**B**) OAT in a homohexameric to monomeric conformation co-migrates with CLPX that also ranges from homohexameric to monomeric positions. As illustration for the readers’ benefit and to complement our complexomics findings with previously known structural information about the interaction between these factors, 3D images were taken from the literature for mtLSU-CP (Extended Data Figure 8g in [71]) and for OAT homohexamers (Figure 8 in [125]), or from public databases for CLPX homohexamer (EMBL-EBI PDBe > 6sfw, last accessed on 7 July 2023) and PLP structure (EMBL-ChEBI 18405, last accessed on 7 July 2023), modifying them to compose this synopsis.

The targets of CLPXP proteolytic activity remain controversial, despite analyses over decades in numerous organisms with diverse technical approaches, most of which involved recombinant overexpression [49,55]. As a novel approach, here, we focused on endogenous proteins in their stable interactions within three mouse tissues, trying to define potential CLPX targets based on four criteria: (1) their co-migration with CLPX in complexome profiles of WT tissues, (2) their co-accumulation with CLPX/VWA8 after CLPP depletion, (3) the appearance at higher molecular weight positions in overlap with CLPX or VWA8 positions after CLPP depletion, and (4) the consistency of such findings between testis, brain, and heart tissues. Preliminary validation experiments confirmed CLPXP dependence for a few selected findings, such as GFM1 accumulation, as a proof of principle that this approach provides valid insights. Within the unbiased complexome profiles of mitochondria, we paid particular attention to the impact of CLPXP (i) on other AAA+ unfoldases to explore their functional overlap, (ii) on the RNA processing and translation machinery in view of previous evidence from bacteria to mice [45,49,53,56,122,123,124], (iii) on mitochondrially translated components of the respiratory chain, and (iv) on matrix enzymes, attempting to elucidate the molecular anomalies at these sites of action. A graphical abstract of the main novel findings is provided in Figure 8 below.

### 3.1. The Putative Role of CLPP-Dependent VWA8 for the mtLSU

The first major observation of our study (Figure 2 and Figure 6) was the CLPP-dependent accumulation and transcriptional induction of the AAA+ unfoldase VWA8. Co-migration of VWA8 was observed with mtLSU-CP intermediate assemblies. On the one hand, these include the L7/L12 stalk factors MPRL19-MRPL12-MRPL53, where GFM1 has its docking site [75] (Figure 8, inset in lower left corner). On the other hand, they include the NPET wall factors, MRPL23-MRPL24 with MRPL65/S30, which accumulate strongly upon CLPP depletion. Their accumulation and distortion may reflect an altered peptide exit channel geometry, which may contribute to the misfolding/aggregation of mitochondrially translated proteins that characterize CLPP-mutant organelles [11,126,127,128], and to the respiratory supercomplex assembly deficits observed in this study. It is important to note that the *Vwa8* transcript is induced in testes already before diplotene pathology occurs, suggesting that VWA8 accumulation is a compensatory effort by cells in response to inadequate CLPX action. The *Clpx* transcript is similarly induced at the earliest testis P17 stage as a potential reaction to functional impairment, but becomes repressed after diplotene meiosis-I failure at the P27 time point, suggesting that excess CLPX over time may acquire a toxic impact.

Very little is known about the role of VWA8 in mitochondria and peroxisomes. VWA8 contains two AAA+ domains that are relatively similar to hexameric dynein-related motors, and a VWA domain towards the C-terminus. In archaea and bacteria, each of the two domains still reside in different proteins, which interact with high frequency. AAA+ domain containing MoxR proteins cooperate with other VWA proteins to insert metal cofactors into substrate molecules [129,130,131]. In *E. coli*, this cooperation is exemplified by the concerted actions of MoxR unfoldase RavA with VWA protein ViaA, which modify cellular sensitivity to aminoglycoside antibiotics (presumably when they bind to bacterial ribosomes), or target the NADH:ubiquinone oxidoreductase-I as a CI homolog, and the fumarate reductase respiratory complex [132,133,134]. RavA is also known for its impact on PLP-dependent lysine decarboxylase, a crucial enzyme for the generation of ornithine-derived polyamines that serve to stabilize RNA structures [135,136,137,138]. It is important to note that the closed ring RavA state bears a close structural similarity to the pseudo-two-fold symmetric crystal structure of bacterial ClpX [139], suggesting a common mechanism or common targets. In *Acidithiobacillus ferrooxidans* bacteria, the MoxR unfoldase CbbQ is upregulated upon iron exposure and acts together with the VWA protein CbbO to rescue the CO2-fixing enzyme Rubisco from inactivity when its cofactor magnesium is absent [140,141].

In a sequence analysis of AAA+ unfoldases across phylogenesis [34], the coexistence of AAA+ and VWA domains within the same protein was first observed in *Synechocystis* cyanobacteria, where the catalytic ChlD subunit of the magnesium chelatase protein complex exerts both functions. It acts to unfold tetrapyrrole ring-containing PPIX and then insert Mg2+ into it, thus being responsible for the first enzymatic step in chlorophyll biosynthesis [142,143,144,145,146]. VWA domains in prokaryotes are known only in magnesium chelatases, or in the cobaltochelatase CobT subunit, which mediates the binding of cobalt during corrin ring formation in the biosynthesis of cobalamin (also known as vitamin B12) [147,148,149]. Given that mammalian mitochondria do not synthesize chlorophyll or cobalamin, what related function could VWA8 fulfil to remain conserved across phylogenesis?

One possibility regarding Mg2+ is its decisive role for DNA/RNA conformation stability and ribosomal subunit cohesion; it controls, for example, the 60-nt GTPase center of 25S rRNA in *E. coli* or the bacterial 5S rRNA loop-E stability within the LSU [150,151,152,153,154,155,156,157]. Another more relevant possibility is the fact that any metalated porphyrin system has a quadrangular shape and binds DNA/RNA avidly when four parallel strands with a high guanine content adopt a G-quadruplex (G4) structure [158,159]. G4 structures control the replication and transcription of rRNA genes and the surface stability of both ribosome subunits [160,161,162]. The interaction of porphyrin variants with G4-RNA structures is among the primary pathogenesis events in some neurodegenerative diseases [163,164]. Metalated porphyrins also have the ability to induce protein aggregation [114]. This second possibility is further supported by the further evolution of VWA functions across phylogenesis. In early eukaryotes, VWA domains become part of intracellular factors that act in DNA repair and ribosomal transport, while metazoan organisms employ VWA domains (also known as the inserted or I-domain in integrins), always in extracellular proteins, to modulate adhesion [147,165]. The MIDAS motif (metal ion-dependent adhesion site) within the VWA domain provided the name for Midasin-1 (known as Rea1 in yeast) as an ancient conserved nuclear AAA-VWA protein with essential functions for cell growth, which was found to make contact with the ribosome separately via the AAA+ ring domain and via the MIDAS/VWA tail, being crucial for the rotation of the L1 stalk and the rotation of the 5S-rRNA/L5/L11 complex, to guarantee the maturation of the pre-60S ribosomal LSU before nuclear export and cytosolic association with the SSU [40,166,167,168].

VWA8, as mitochondrial protein that combines AAA+ and VWA domains, therefore possibly functions like its prokaryotic ancestors, unfolding porphyrin or corrin rings to insert chelated magnesium or another cationic metal [169]. This notion is compatible with excess CLPX affecting heme biosynthesis and several heavy metals accumulating with VWA8 in PRLTS3 pathology. VWA8 is localized at the matrix side of the mitochondrial inner membrane [170], and iron-chelated heme is produced inside the inner membrane in close proximity to the MICOS complex [171]. VWA8 depletion in human and zebrafish causes developmental deficits with microcephaly [172]. Heme is not only the crucial component of hemoglobin in blood cells, but also the key cofactor of cytochromes, e.g. in the respiratory chain [173,174]. Complex IV in particular, is assembled depending on the availability of heme, copper, zinc, and magnesium [175,176,177,178]. This knowledge may explain the association of VWA8 with respiratory chain components (see Figure S1) and its impact there, but why would VWA8 associate selectively with the mtLSU L7/L12 stalk and NPET? The prokaryotic L7/L12 stalk is also known as the GTPase-associated center (GAC), where the translation elongation GTPases EF-G (orthologous to GFM1), IF2, RF3, and LepA dock and acquire full activity [179]. It is known that the decision as to whether ribosomes disassemble or, alternatively, inter-subunit bridges become stabilized for ribosome recycling is strongly dependent on magnesium concentrations and on EF-G, which contains magnesium in its activated GTPase site [180]. At this GAC, the paused translation of polyproline tracts can be rescued with the help of polyamines [137]. Regarding the association of VWA8 with the NPET, it is interesting to note that recent evidence shows the formation of G4-rRNA structures to bind heme to such an extent that this determines heme bioavailability [181,182]. The elevated abundance of such G4 structures in CLPP-null mitoribosomes is likely, because of the observed accumulation of the G4-RNA-binding protein GRSF1 there [56]. Thus, our observation that VWA8 may interact with mtLSU proteins and with respiratory complexes is indeed compatible with current knowledge. In previous studies of mouse hepatocytes, VWA8 depletion was found to cause elevated respiratory chain complex abundance and activity, together with oxidative stress and increased protein turnover, while non-canonical gamma-amino butyric acid (GABA) levels decrease [183,184]. Thus, both experimental findings and data mining provide plausible evidence that VWA8 acts downstream from CLPX, both targeting the mitoribosome and respiratory complexes, likely acting within the homeostasis of PLP-mediated unconventional amino acids (like ALA and ornithine) and their derivatives like heme and polyamines, and the homeostasis of cationic metals, all of which are important as RNA interactors [137,182].

### 3.2. The Putative Role of CLPX for the mtLSU Intermediate Assembly

The second major observation of our study (Figure 3 and Figure 7) was the physiological co-migration of CLPX itself with other mtLSU-CP intermediate assemblies, a completely novel finding. Upon pathological CLPP absence, only specific mtLSU proteins show significant accumulation, whereas practically all mtSSU factors accumulate strongly without selectivity and without CLPX co-migration (Table S1). The selective impact of CLPXP on mtLSU was surprising, because Perrault syndrome is triggered by mutant HARS2/LARS2/ERAL1, all of which are associated with the mtSSU. In addition, progressive sensorineural deafness due to the chronic administration of aminoglycoside antibiotics is also caused by neurotoxic interference and genetic variance at the mtSSU [185,186,187]. A detailed review of mitoribosomal pathology underlying progressive deafness, as in PRLTS, was recently compiled [49]. It should also be noted that the principal target of the CLPX cofactor, PLP, at the bacterial SSU is the inhibition of mRNA-dependent aminoacyl-tRNA binding [118]. Thus, mtSSU pathology is widespread and massive, but may not be a direct CLPXP effect, instead occurring as consequence of tRNA processing problems. The selective impact of CLPXP on mtLSU intermediate assemblies was also unexpected, because the previous concept assumed that CLPXP acts on complete ribosomes when they stall during translation. In contrast, the complexome profiles suggest that CLPX associates with specific subcomplexes, either during mtLSU assembly or after mtLSU disassembly.

The CLPXP-relevant intermediate assemblies of the mtLSU have been described before [64,65,66,67,68,69], and the stress-dependent redistribution of the mitochondria-specific MRPL18-L38-L40-L46-L48-L55 cluster in complexomics profiles was reported for Barth syndrome as a consequence of mitochondrial cardiolipin anomalies [65,67,68]. This subcomplex of the central protuberance contributes to the connection between mtLSU and mtSSU, via inter-subunit bridges mB1a and mB1b from mL40-mL46-mL48 in the mtLSU to the mtSSU mitochondria-specific GTP-binding component mS29 (also abbreviated as DAP3, for Death-Associated Protein 3) [188,189]. Within this mtLSU cluster, MRPL55 (=bL31m), MRPL18 (=uL18m), MRPL38, and MRPL46 show the largest accumulation upon CLPP absence, and MRPL18 with MRPL38 exhibit the most dispersion in BNE gels, in perfect overlap with CLPX dispersion.

It was unexpected that a protein cluster that did not exist in bacteria but instead arose in mitochondria should be the target of ancient CLPXP. It is therefore important to note that this MRPL18-associated protein cluster serves in bacteria as a docking site for 5S rRNA, but in mitochondria, the cluster was modified to bind tRNA^Val^ or tRNA^Phe^ [73]. Although the peptidyltransferase activity of bacterial LSU is fulfilled by 23S rRNA in a tight interaction with magnesium microclusters [190], an indispensable addition occurred during phylogenesis with 5S rRNA and its associated proteins, using allosteric effects to stabilize and coordinate various centers of the ribosome and linking their activity with cell proliferation [191,192,193,194]. Recent observations suggested that 5S rRNA in mammalian cells can be sequestered from cytosolic ribosomes into mitochondria via interactions with proteins MRPL18 and rhodanese, where 5S rRNA can occupy its ancestral position at the mtLSU central protuberance instead of tRNA^Val/Phe^ [195,196,197], but it is unclear if this finding is reproducible. Thus, the assembly of any protein with 5S-rRNA in bacteria and with tRNA^Val/Phe^ in mammalian mitochondria—such as MRPL18/38—might need CLPXP.

While it is unclear what actions would be performed at this MRPL18-associated cluster, it is noteworthy that PLP as a CLPX cofactor was reported to act at the bacterial LSU, dissociating the two subunits and inhibiting translation elongation, via the modification of the 5S rRNA binding proteins L5-L18-L25, stalk protein L1, and the elongation GTPase GFM1 ortholog [118]. Also the VWA8-co-migrating ribosomal L7/L12 stalk proteins are targets of PLP modification [118]. Overall, the data raise the possibility that CLPXP with PLP as a cofactor act on this mitochondria-specific cluster to control associations/disassociations of subcomplexes within the mtLSU, and of inter-subunit bridges to the mtSSU. In addition, CLPXP via PLP might modulate translation efficiency via the tRNA^Val/Phe^-associated cluster.

The CLPX-co-migrating stalk proteins MRPL1 and MRPL9 show even stronger accumulation upon CLPP deficiency, so how would they connect with such a scenario? As mentioned, L1 is a target of CLPX-cofactor PLP [118]; the maturation of the L1 stalk involves a position shift during assembly steps mediated by mt-ACP2 (NDUFAB1); and the flexibility of the mature L1 stalk contributes to the translocation of tRNAs during translation [72,198]. Thus, the excess of MRPL1-L9 abundance also adds to the evidence that mutant CLPXP has an impact on translation at the elongation step.

Our demonstration that CLPX interacts with MRPL38 at endogenous abundance levels in co-immunoprecipitation assays indicates that the function of CLPXP for mtLSU assembly occurs with similar abundance, as the previously observed effects of recombinant CLPX for GFM1 control in translation elongation, and as the ALAS/OAT control in the metabolism of non-classical amino acids.

### 3.3. Potential Functions of CLPX and VWA8 for Specific Translation/RNA Granule Factors, Matrix Enzymes, and PLP, as Well as Chaperones

A third relevant finding of our study (Figures S2 and S3) included the co-migration of CLPX in WT tissue with additional specific translation factors and matrix enzymes. Among the ribonucleoproteins, the histidine-tRNA synthetase HARS2, the elongation GTPase GFM1, and the cytosolic translation initiation core component EIF3C showed significant accumulation in CLPP-null tissue. HARS2 is obviously the most important result given that its mutation causes the same phenotype as CLPP deficiency, the autosomal recessive Perrault syndrome. HARS2 localizes in the mitochondrial RNA granule and associates with the mtSSU, representing a bridge between the pathology observed there and CLPXP. It is not known to associate with PLP in a specific manner, but the principal target of PLP at the bacterial SSU is the inhibition of mRNA-dependent aminoacyl-tRNA binding in general [118]. Indeed, other mitochondrial amino acid-tRNA ligases also accumulate in CLPP-null tissue (in testis, the increase in DARS2, EARS2, FARS2, GARS, KARS, NARS2, SARS2, TARS2, VARS2, and YARS2 abundance is also >2-fold), although their physiological co-migration with CLPX and their dispersion in CLPP-null tissue are not as clear as for HARS2. Previous CLPXP target surveys throughout phylogenesis have already identified GFM1 as the most consistent result [49], and a mouse CLPXP substrate-trap screening using CLPPWT-FLAG versus CLPPS149A-FLAG plasmids transfected into CLPP-null murine embryonic fibroblasts also identified GFM1 (named EFG1 in this report) as one of the two most credible candidates [45]. We now confirm that this interaction between CLPX and GFM1 occurs at endogenous abundance levels in co-immunoprecipitation assays, even in WT tissues, and thus has physiological significance.

The mitochondrial RNA granule components PTCD1, LRPPRC, and SLIRP are clearly accumulated in CLPP-null tissue, but PTCD1 was not detectable in WT complexome profiles, so its physiological CLPX co-migration remains unclear. LRPPRC migrates with SLIRP well above the physiological CLPX migration range, so their association with CLPX may occur only during pathogenesis. Thus, the RNA granule might become involved in Perrault syndrome only via the off-target effects of excess CLPX.

The matrix enzyme homeostasis, accumulation, and CLPX co-migration of PLPBP in CLPP-null testis (severely affected in PRLTS3) but not MEFs (spared in PRLTS3) (Figure S3 and Figure 5), as matrix protein responsible for the homeostasis of PLP, apparently contribute to ALAS2 co-accumulation (see also Table S1) and increased heme production with elevated tissue iron levels. As seen in Figure 6, the mRNA for PLPBP is progressively induced during testis aging, suggesting that PLP also accumulates over time. PLP is an ancient factor that has existed since the prebiotic RNA world [199], which evolved as a chaperone [31] and cofactor of enzymes that mediate transamination and other reactions such as desulfurization [200]. It is crucial for the non-classical biosynthesis of unconventional amino acids [201], e.g., when an additional amino group is placed within an amino acid at the gamma position or delta position from the carboxy-acid group [202]. Thus, it is essential for the generation of delta-amino levulinic acid (ALA) by ALA-synthase [203], and of ornithine by ornithine-delta-aminotransferase (OAT) [204]. It is also crucial for cysteine desulfurase in the biosynthesis of molybdopterin [205], or in the FeS-cluster-mediated assembly of respiratory supercomplexes [206]. Delta-amino acids serve as building blocks in the biosynthesis of porphyrin rings (ALA), corrin rings (ALA), and polyamines (ornithine), while molybdopterin serves in the biosynthesis of the molybdenum cofactor MoCo. On these platforms, heavy metals can be bound stably and harnessed for metabolic functions—the porphyrin chelation of iron produces heme, the corrin binding of cobalt generates cobalamin, and molybdopterins stabilize molybdate. Polyamines provide heavy metal tolerance [207]. Interestingly, the incorporation of manganese into mitochondrial superoxide dismutase was also shown to depend on PLP. This occurs in a Mtm1p-dependent process in yeast, and PLP bioavailability in mitochondria depends on Mtm1p [208]. The mischarging of tRNA-Lys with ornithine occurs permanently in cells and has to be corrected via surveillance editing [209]. Its misincorporation into polypeptide chains induces a cyclic structure and translation arrest with cell toxicity [210]; therefore, the cleavage of such aggregation-prone fragments in mitochondria might be a function of CLPP. Jointly, these lines of evidence suggest that CLPX and PLP act together in the biosynthesis of several non-classical amino acids that are needed as building blocks for the docking sites of heavy metals. Therefore, the neurodegeneration in PRLTS3 may reflect a gain-of-function of CLPX and of its cofactor PLP in affected tissues. Conversely, neonatal epilepsy and chronic encephalopathy due to PLPBP or PNPO loss-of-function mutations benefits from therapeutic PLP substitution [211].

In this context, it is noteworthy that the CLPX-co-migrating ALDH18A1 protein multimer (also known as Glutamate-5-SemiAldehyde Synthetase or as Pyrroline-5-Carboxylate Synthetase = P5CS, an ortholog of bacterial ProA) also accumulates upon CLPP absence, and was previously reported to be affected by the CLPX cofactor PLP [212]. Proline, as the most frequent cause of ribosomal stalling, is metabolically derived from glutamate via Glutamate γ-SemiAldehyde (GSA) and its cyclization product Δ1-Pyrroline-5-Carboxylate (P5C) [213]. ALDH18A1 co-accumulates in CLPP-null tissues with Ornithine delta Amino Transferase (OAT) [56,57], another proline homeostasis regulator, which is known to depend on PLP as a cofactor to interconvert GSA and ornithine as an intermediate enzymatic step between proline and the urea cycle [119,120]. OAT is the mammalian ortholog of bacterial HemL (Glutamate-1-Semialdehyde-2,1-AminoMutase = GSAM), which has a remarkable three-dimensional homology with ALAS and contributes to the production of ALA, with the subsequent generation of tetrapyrrole rings in the heme/chlorophyll biosynthesis pathway of bacteria and plants [214,215,216]. OAT was proposed as a CLPXP substrate in a previous study, where a substrate-trap assay overexpressing CLPPWT-FLAG versus CLPPS129A-FLAG plasmids in CLPP-null MEFs was combined with an N-terminomic analysis of mouse heart proteome profiles [57]. Our demonstration that CLPX interacts with OAT at endogenous abundance levels in co-immunoprecipitation assays of WT (and more strongly in CLPP-null tissue) indicates that not only ALAS association with PLP is controlled by CLPX, but a similar control mechanism is credible for OAT and its metabolic function. Our finding that *Aldh18a1* and *Oat* mRNA are induced in testes before pathology occurs may be interpreted as cellular efforts to compensate a mis-assembly of the OAT homohexameric ring with PLP, with the consequent impairment of its metabolic function in the interconversion of P5C and ornithine. These observations highlight similarities between proline and porphyrin biosynthesis, both starting with the conversion of GSA to cyclic pyrrole, in PLP- and CLPX-dependent pathways.

The accumulation of the molecular chaperones TRAP1 and GRP75 in the mitochondrial matrix of CLPP-null tissues may simply be due to the altered assembly and slowed turnover of ribonucleoprotein complexes as a secondary phenomenon. Furthermore, their apparent co-migration with CLPX may simply be a coincidence, given that both have a molecular weight around 70–75 kDa and their interaction with target proteins would make them migrate within the range of CLPX position dispersion. Thus, they may be purely downstream players in Perrault syndrome pathogenesis. However, it is relevant to be aware that the stress-induced cytosolic isoform of MRPL18 alters the abundance of heat shock proteins (HSPs) at the level of translation, acting via its 5S rRNA or tRNA^Val/Phe^ association [217]. The 5S rRNA was also reported to contribute to the folding of RNA-targeting members of the HSP70 chaperone family such as DnaK in *E. coli*, the ortholog of mammalian HSPA9 in mitochondria [218]. An accumulation of the tetrapyrrole precursors of heme is also a known inducer of molecular chaperones of the HSP70 family [219]. The impact of CLPXP on MRPL18 abundance and distribution, with downstream consequences for the subcellular availability of tRNA^Val/Phe^, jointly with the impact of CLPXP on the heme biosynthesis pathway, may therefore conspire to modify the stress adaptation of mitochondria and their eukaryotic hosts via molecular chaperones as an early pathogenesis event. It is important to note the high selectivity of these chaperone accumulations that constitute a pathway in themselves. As in bacteria, a conserved interaction persists between CLPX and the HSP70 family member HSPA9 with its co-chaperones GRPEL1-GRPEL2-DNLZ-CCDC127, so presumably, they share specific target protein clusters.

### 3.4. Potential Functions of CLPX and VWA8 for Specific Respiratory Chain Complex Assembly

A fourth relevant finding concerns the question as to whether folding problems or translation problems are primary. It is well known that (1) CLPXP mainly targets the mitoribosome, (2) CLPP mutation leads to translation fidelity deficits, (3) translation stalls mainly upon the synthesis of polyproline motifs, (4) a triproline motif occurs only in mtCO1 among all mitochondrially translated factors, and (5) the corresponding complex IV activity shows the strongest reduction within the respiratory chain in CLPP-null mouse tissues. Thus, the decreased MTCO1 abundance to 30% (Figure 4) was not surprising, but the failure of assembly to respiratory supercomplexes was even stronger. Given that none of the mitochondrially translated factors combine CLPX co-migration with accumulation in the complexomics profiles (Figure S4), they are presumably affected indirectly and do not represent CLPXP targets. Two assembly factors, named COX15 and SFXN4, whose accumulation and CLPX co-migration is prominent, might explain the assembly and turnover anomalies observed for complex IV and complex I, respectively. COX15 (also known as heme-A synthase) localizes to the inner mitochondrial membrane, where it cooperates with ferredoxin and ferredoxin reductase to convert heme-O to heme-A, which is required for the proper folding of the COX1 subunit and subsequent respiratory complex IV assembly [104,105,107,220,221,222]. Mutations in COX15 trigger a complex IV deficit with subsequent infantile cardioencephalopathy that resembles Leigh syndrome [223]. Possibly explaining the complex I deficits, SFXN4 is descended from a large family of iron transporters, impacts cytosolic iron homeostasis via the IRP1-aconitase switch, affects iron redistribution between cytosol and mitochondria, and impacts mitochondrial Fe-S clustering and heme biosynthesis, assisting the MCIA complex in the assembly of the respiratory ND2 module and influencing the levels of ferrochelatase and ALAS2 [100,224,225]. Its mutation triggers phenotypes of macrocytic sideroblastic anemia, weight deficits, and neurological impairments [226]. Overall, the supercomplex assembly in the respiratory chain is more affected than the translation of individual proteins such as MTCO1, and the underlying protein-folding problem may be reflected in the accumulation of COX15 and SFXN4, presumably due to altered homeostasis, firstly of CLPX/PLP (despite compensatory VWA8 induction) and secondly of iron/heme in the mitochondrial matrix upon CLPP absence.

## 4. Materials and Methods

### 4.1. Animal Breeding, Aging, and Dissection

The generation of CLPP-null (*Clpp*^−/−^) mice has been described in detail before [51], and pups were bred from heterozygous mating. The mice were housed under specific-pathogen-free conditions under a 12 h light cycle with food and water ad libitum in the central animal facility (ZFE) of the University Hospital Frankfurt. This study was conducted according to the guidelines of the Declaration of Helsinki and approved by the Institutional Review Board of Regierungspräsidium Darmstadt (protocol code: V54-19c20/15-FK/1073, date of approval: 28 September 2016). Due to the complete infertility of CLPP-null homozygous mice of both sexes, breeding was performed simultaneously among multiple pairs of heterozygous mutation carriers, aging them under identical conditions until they were sacrificed for analysis. WT and CLPP-null mutant mice aged to 8 weeks were sacrificed by cervical dislocation and used for organ dissection. Tissues were snap-frozen in liquid nitrogen and stored at −80 °C.

### 4.2. Experimental Design and Statistical Rationale

Given that complexome investigations are laborious and mass spectrometry core facilities have to serve many collaborating teams, we had to process a minimal number of samples in the survey, but later used many additional samples in validation experiments. Since the consistency between different tissues provides valuable insights, we decided to use 1 WT versus 1 CLPP-null mouse, comparing the brain, heart, and testis, resulting in 3 control versus 3 mutant samples. The results were not analyzed in a statistical manner, but the segregation of protein complexes, supercomplexes, and assembly intermediates was investigated based on consistency criteria.

### 4.3. Isolation of Mitochondria in Sucrose Gradients

Mitochondria were resuspended in a solubilization buffer (50 mM imidazole, pH 7.50, 50 mM NaCl, 1 mM EDTA, 2 mM aminocaproic acid) and solubilized with 20% (*w*/*v*) digitonin (Serva, Heidelberg, Germany). The samples were supplemented with 2.5 µL of 5% Coomassie G250 in 500 mM aminocaproic acid and 5 µL of 0.1% Ponceau-S in 50% glycerol. Equal protein amounts of the samples were loaded on top of a 3 to 18% acrylamide gradient gel (dimension: 14 × 14 cm). After native electrophoresis in a cold chamber, blue native gels were fixed in 50% (*v*/*v*) methanol, 10% (*v*/*v*) acetic acid, 10 mM ammonium acetate for 30 min and stained with Coomassie (0.025% Serva Blue-G, 10% (*v*/*v*) acetic acid) [227].

### 4.4. Sample Preparation for Complexome Profiling

Each lane of a BNE gel was cut into equal fractions and collected in 96-well filter plates (30–40 µm PP/PE, Pall Corporation, Port Washington, NY, USA). The gel pieces were destained in 60% methanol and 50 mM ammonium bicarbonate (ABC). The solutions were removed via centrifugation for 2 min at 600× *g*. the proteins were reduced in 10 mM DTT and 50 mM ABC for one hour at 56 °C and alkylated for 45 min in 30 mM iodoacetamide. The samples were digested for 16 h with trypsin (sequencing grade, Promega, Madison, WI, USA) at 37 °C in 50 mM ABC, 0.01% Protease Max (Promega), and 1 mM CaCl_2_. Peptides were eluted in 30% acetonitrile and 3% formic acid, centrifuged into a fresh 96-well plate, dried in a speed vac, and resolved in 1% acetonitrile and 0.5% formic acid.

### 4.5. Setting HPLC/MSMS Methods

Liquid chromatography/mass spectrometry (LC/MS) was performed on a Thermo Scientific™ Q Exactive Plus equipped with an ultra-high performance liquid chromatography unit (Thermo Scientific Dionex Ultimate 3000) and a Nanospray Flex Ion-Source (Thermo Scientific, Waltham, MA, USA). The peptides were loaded on a C18 reversed-phase precolumn (Thermo Scientific) followed by separation on a 2.4 µm Reprosil C18 resin (Dr. Maisch GmbH, Ammerbuch, Germany) in-house packed PicoTip emitter tip (diameter: 100 µm, 15 cm, from New Objectives, Littleton, MA, USA) using a gradient from 4% ACN, 0.1% formic acid to 25% eluent-B (99% acetonitrile, 0.1% formic acid) for 30 min, followed by a second gradient to 50% B with a flow rate of 300 nL/min and washout with 99% B for 5 min. MS data were recorded via data-dependent acquisition. The full MS scan range was 300 to 2000 *m*/*z,* with a resolution of 70,000 and an automatic gain control (AGC) value of 3E6 total ion counts, with a maximal ion injection time of 160 ms. Only higher charged ions (2+) were selected for MS/MS scans with a resolution of 17,500, an isolation window of 2 *m*/*z,* and an automatic gain control value set to E5 ions, with a maximal ion injection time of 150 ms. MS1 data were acquired in profile mode.

### 4.6. Data Analysis with MaxQuant

The MS data were analyzed with MaxQuant (v1.6.17.0) [228] using the default settings. The search engine was integrated in MaxQuant. Proteins were identified using the mouse reference proteome database UniProtKB with 55,470 entries, released in April 2021. The enzyme specificity was set to Trypsin, with a maximal number of missed cleavages of 2. Acetylation (+42.01) at the N-terminus and the oxidation of methionine (+15.99) were selected as variable modifications and carbamidomethylation (+57.02) as fixed modification on cysteines. The mass tolerance for precursor and fragment ions was 4.5 ppm. The false discovery rate (FDR) for the identification protein and peptides was 1%. Intensity-based absolute quantification (IBAQ) values were recorded. Reverse hits and contaminants were excluded. The sum of all IBAQ values of the datasets was normalized to the corresponding control set. Protein abundance within native lanes was normalized to the maximum appearance to enable the comparison of mitochondrial complexes between the controls and mutants. All primary data were made publicly available by the ProteomeXchange Consortium via the PRIDE [229] partner repository with the dataset identifiers PXD035352 (testis), PXD036901 (brain), and PXD036933 (heart). The entries contain the individual peptide sequences, single peptide identifications, all modifications observed, peptide identification scores, accession numbers, peptide assignments, and quantification measurements.

### 4.7. Complexome Profiling for OXPHOS Subunits and Other Mitochondrial Assemblies

The slice number of the maximum appearance of the mitochondrial CI (979,577 Da), CII (122,945 Da), CIII dimer (483,272 Da), CIV (213,172 Da), CV (537,939 Da), and respiratory supercomplexes containing CI, III dimer, and one copy of CIV (1,676,021 Da) was used for native mass calibration. The software NOVA (v.0.5.7) was used for the hierarchical clustering of the complexomics data [230].

### 4.8. Protein–Protein Interaction Bioinformatics and Pathway Enrichment Analysis via STRING

To survey the known protein associations of the poorly studied AAA+ ATPase, data were extracted from the biomedical interaction repository BioGrid (https://thebiogrid.org/, last accessed on 8 November 2021), where endogenous VWA8 was detected in a complex with bait proteins. The STRING webserver (https://string-db.org/, last accessed on 8 November 2022) was used for visualization and statistics on these protein–protein interactions and pathway analyses. Filtered gene symbols were entered into the multiple proteins option for *Mus musculus,* and the graphical output was archived, highlighting factors with identical features by color, as explained in the figure caption.

### 4.9. Heavy Metal Analysis

The heavy metal abundance was measured in the brain hemispheres (10 WT versus 10 CLPP-null) of 4-month-old mice as described previously [231]. In brief, the frozen mouse brain samples were thawed at room temperature and transferred in total into a digestion tube. Dry matter quantity was determined after oven-drying at 104 °C for 4 h. Due to the small amount of material, the dry matter could only be determined as a single measurement. Dried samples were subsequently mineralized through microwave digestion (Mars6, CEM GmbH, Kamp-Lintfort, Germany) using 3 mL of nitric acid, 1.5 mL of hydrogen peroxide, and 2.5 mL of water. The digested samples were analyzed for Ca, Co, Cu, Fe, Mg, Mn, Mo, Se, Vn, and Zn via inductively coupled plasma mass spectrometry (ICP-MS; PerkinElmer, Waltham, MA, USA), whereby indium and germanium were used as the internal standards and commercial standard solutions for calibration.

### 4.10. Quantitative Immunoblots

Protein was isolated with RIPA buffer (50 M Tris/HCl pH 8.0, 150 mM NaCl, 0.1% SDS, 1% Triton X-100, 0.5% sodium deoxycholate, 2 mM EDTA, and protease inhibitor cocktail from Sigma Aldrich, St. Louis, MO, USA). The protein content was determined using the BCA assay (Life Technologies, Carlsbad, CA, USA); 15 µg of protein was loaded for quantitative immunoblotting. Subcellular fractionations in mouse embryonic fibroblasts (MEFs) (3 WT versus 3 CLPP-null) were obtained as described previously [232]. Subcellular fractionations from tissues were obtained as described in [233]. Primary antibodies used were as follows: CLPP (Proteintech, 15698-1-AP, 1:1000), CLPX (Abcam – Cambridge, UK, ab168338, 1:1000), VWA8 (Invitrogen, PA5-58648, 1:1000), PLPBP (Proteintech, 25154-1-AP, 1:1000), HSPA9 (Oxford Biomedical Research–Rochester Hills, MI, USA, GR 02, 1:1000), TRAP1 (Abcam, ab109323, 1:1000), COX15 (Proteintech, 11441-1-AP, 1:1000), PTCD1 (Abclonal—Woburn, MA, USA, A16219, 1:1000), MRPL38 (Proteintech, 15913-1-AP, 1:1000), MRPL18 (Novus–St. Louis, MO, USA, NBP2-94482, 1:1000), GFM1 (Proteintech, 14274-1-AP, 1:1000), OAT (Invitrogen, PA5-92842, 1:1000), ALDH18A1 (Proteintech, 17719-1-AP, 1:1000), HSP60 (Santa Cruz Biotechnology—Dallas, TX, USA, sc-13115; 1:500), and GAPDH (Calbiochem—Burlington, MA, USA, CB1001; 1:1000). Secondary antibodies (1:15,000) were fluorescence-labeled, obtained from Li-Cor Biosciences (Lincoln, NE, USA), and either anti-mouse or anti-rabbit. The fluorescent signals were detected using the Li-Cor Odyssey Classic Instrument and were densitometrically analyzed with Image Studio Lite version 5.2 (Li-Cor Biosciences).

### 4.11. RT-qPCR

Total RNA was isolated from testis tissues (3 WT versus 3 CLPP-null) with TRI reagent (Sigma-Aldrich), and reverse transcription was performed with the SuperScript IV VILO Master Mix (Thermo Fisher Scientific). Reverse-transcriptase real-time quantitative polymerase chain reaction (RT-qPCR) was carried out with TaqMan^®^ Gene Expression Assays (Thermo Fisher Scientific) in complementary deoxyribonucleic acid (cDNA) from 10 ng of total RNA in 10 μL reactions with 2× Master Mix (Roche, Basel, Switzerland) in a StepOnePlus Real-Time PCR System (Applied Biosystems, Waltham, MA, USA). The data were analyzed with the 2−ΔΔCT method [234]. The following Taqman assays were applied: *Aldh18a1*: Mm00444767_m1, *ClpP*: Mm00489940_m1, *ClpX*: Mm00488586_m1, *Cox15*: Mm00523096_m1, *Gfm1*: Mm00506856_m1, *Hspa9*: Mm00477716_g1, *Mrpl18*: Mm00775800_g1, *Mrpl38*: Mm00452473_m1, *Oat*: Mm00497544_m1, *Plpbp*: Mm00475293_m1, *Ptcd1*: Mm00505236_m1, *Sfxn4*: Mm00446462_m1, *Trap1*: Mm00446003_m1, *Tbp*: Mm00446973_m1, *Vwa8*: Mm01324241_m1.

### 4.12. Co-Immunoprecipitation

The tissues were lysed in a lysis buffer (20 mM Tris/HCl pH 8.0, 137 mM NaCl, 2 mM EDTA, 1% NP40, 1% glycerol with Protease-Inhibitor Cocktail cOmplete (Roche)) via 30 min head-to-head rotation at 4 °C. Nuclear debris was removed via centrifugation at full speed at 4 °C for 20 min. The protein content was determined via BCA (Life Technologies). A total of 1000–1500 µg of protein lysate was incubated with 5 µg of pull antibody and rotated for 2 h at room temperature (RT). In the meantime, 1.5 mg of Dynabeads (Thermo Fisher, #10004D) was washed 3× with PBS/T and added to the lysate/antibody solution. The mix was rotated head-to-head for 60 min at RT. After 5 washes with PBS, the tubes were fixed on a magnetic stand and elution was carried out with 40 µL of 50 mM glycine, pH 2.8, and the eluate was mixed with a loading buffer, boiled for 5 min at 90 °C, and loaded for SDS electrophoresis. The antibodies used for CoIP were CLPX (Invitrogen, PA5-79052), CLPX (Abcam, ab168338), CLPX (NSJ Bioreagents, RQ5618), CLPP (Proteintech, 15698-1-AP), MRPL38 (Invitrogen, PA5-118074), GFM1 (Proteintech, 14274-1-AP), and OAT (Invitrogen, PA5-92842).

### 4.13. Statistics

Statistical analyses of the quantitative immunoblot and RT-qPCR results were conducted by using GraphPad Prism (version 8.4.2, GraphPad), using unpaired Student’s t-tests with Welch’s correction. The results, including the standard error of the mean (SEM) and *p*-values, were visualized in bar graphs, with the following significances illustrated with asterisks: * *p* < 0.05; ** *p* < 0.01; *** *p* < 0.001; **** *p* < 0.0001; T (statistical trend) 0.05 < *p* < 0.1.

## 5. Conclusions

Jointly, the analyses of the protein complexome profiles in mitochondria from three tissues in WT versus CLPP-null mice regarding accumulation, CLPX/VWA8 co-migration, and dispersed gel positions have elucidated the interaction between these endogenous AAA+ unfoldases and their targets. The results highlight assembly problems at mtLSU, RNA granules, the respiratory chain complexes I and IV, and unconventional amino acid homeostasis enzymes, with the consequent accumulation of molecular chaperone proteins. Practically all protein accumulations appeared to represent secondary compensatory events, presumably due to assembly and function deficits; only the CLPX protein accumulation led to a compensatory transcriptional downregulation and may therefore by itself explain PRLTS3 pathology through its toxic gain-of-function. The data provide proof-of-principle that CLPX controls not only the access of PLP to ALAS2 to enhance its activity, but similarly OAT, GFM1 and MRPL38 may be modulated in this way. This provides mechanistic detail in the understanding of PRLTS3 pathogenesis where CLPX is in excess, and progressive deafness is due to mitoribosomal translation problems.

## Figures and Tables

**Table 1:** Accumulation of several heavy metals in CLPP-null brain. Quantification was carried out in 10 WT versus 10 CLPP-null brains at the age of 4 months via ICP-MS. Values are given in mean ± standard error per dry matter of tissue. * *p* < 0.05; **** *p* < 0.0001.

Elements, ppm	WT	CLPP-Null	Fold Change	*p*-Value	Significance
Iron/Ferrum (Fe)	83.33 ± 2.82	113.85 ± 3.60	1.366	<0.0001	****
Molybdenum (Mo)	221.48 ± 9.10	299.04 ± 7.04	1.350	<0.0001	****
Cobalt (Co)	37.04 ± 1.74	44.98 ± 2.46	1.214	0.0178	*
Manganese (Mn)	1.47 ± 0.09	1.76 ± 0.07	1.193	0.0235	*

## Data Availability

All primary data were made publicly available by the ProteomeXchange Consortium via the PRIDE partner repository with the dataset identifiers PXD035352 (testis), PXD036901 (brain), and PXD036933 (heart).

## Supplementary Materials

The supporting information can be downloaded at https://www.mdpi.com/article/10.3390/ijms242417503/s1.

## Abbreviations

12S rRNA	ribosomal RNA component of SSU in bacteria
16S rRNA	ribosomal RNA component of LSU in bacteria
16S rRNA	ribosomal RNA component of mtSSU in mammals
23S rRNA	ribosomal RNA component of mtLSU body
28S	mtSSU of mammalian mitoribosomes
39S	mtLSU of mammalian mitoribosomes
55S	completely assembled mitoribosome
5S rRNA	ribosomal RNA component of the mtLSU central protuberance
AAA+	protein superfamily of ring-shaped P-loop NTPases
ABC	Ammonium Bicarbonate
ACAD9	Acyl-Coenzyme A Dehydrogenase Family, Member 9, MCIA component
ACN	Acetonitrile
ACTB	beta-Actin protein
AFG3L2	ATPase Family Member 3-Like 2, Matrix AAA Peptidase Subunit 2
AGC	Automatic gain control
ALA	delta-Amino Levulinic Acid
ALAS	5-AminoLevulinic Acid Synthase
ALAS2	Delta-Amino-Levulinic-Acid-Synthase-2, erythroid-specific, mitochondrial
ALDH18A1	P5CS, Aldehyde Dehydrogenase 18 Family Member A1
ATAD3	ATPase Family AAA Domain-Containing Protein 3
ATPase	Adenosine 5’-TriPhosphatase
BCA	Bicinchoninic Acid
BCS1L	Ubiquinol-Cytochrome C Reductase Complex Chaperone
BNE	Blue Native gel Electrophoresis
CaCl2	Calcium chloride
Cbb	Prokaryotic Rubisco activation protein
CbbQ	Prokaryotic Nitric oxide reductase NorQ protein; AAA ATPase
cDNA	complementary Deoxyribonucleic Acid
ChlD	Prokaryotic Magnesium protoporphyrin IX chelatase, d subunit
CI	Respiratory Complex I
CII	Respiratory Complex II
CIII	Respiratory Complex III
CIV	Respiratory Complex IV
CCDC12	Coiled-Coil Domain-Containing Protein 127
ClpA	Prokaryotic ATP-dependent specificity component of ClpAP protease
CLPB	Caseinolytic peptidase B protein homolog; AAA+ ATPase
CLPP	ATP-dependent Clp protease Proteolytic subunit, mitochondrial
CLPX	specificity component of Clp protease complex, AAA+ ATPase
CLPXP	proteolytic machine with components CLPP and CLPX
CO2	Carbon dioxide
CobT	Prokaryotic Cobaltochelatase
CoIP	Co-immunoprecipitation
*Cox1*	Cytochrome-c-Oxidase subunit 1, catalytic
COX15	Cytochrome-c-Oxidase assembly factor; acts in heme-A biosynthesis
CP	Central Protuberance of mtLSU
CV	Respiratory Complex V
DNA	Deoxyribonucleic Acid
DnaJ	Prokaryotic co-chaperone Hsp40; acts with DnaK/GrpE in disassembly
DnaK	Prokaryotic chaperone Hsp70
DAP3	MRPS29, Death Associated Protein 3
DNLZ	DNL-type Zinc-finger Protein; mtHsp70-escort protein
DTT	Dithiothreitol
ECSIT	Evolutionary Conserved Signal Intermediate In Toll pathway, mitochondrial
EDTA	Ethylenediaminetetraacetic Acid
EF-G	Translation Elongation Factor G, ortholog of GFM1
EIF3	Eukaryotic translation Initiation Factor 3 protein complex
EIF3C	Eukaryotic translation Initiation Factor 3, subunit C
ERAL1	Era-Like 12S mitochondrial rRNA chaperone 1
FASTKD4	TBRG4, FAST Kinase Domain-Containing Protein 4
FC	Fold Change
FDR	False Discovery Rate
FECH	Ferrochelatase, inserting iron into porphyrin rings to produce heme
Fe-S cluster	Iron–Sulfur cluster
FLAG	Octapeptide tag for proteins, with sequence DYKDDDDK
G4	Guanosine-rich quadruplex structure of DNA/RNA
GABA	Gamma-Aminobutyric Acid, main inhibitory neurotransmitter
GAC	GTPase-AssociatedC
GAPDH	Glyceraldehyde 3-Phosphate Dehydrogenase, glycolysis enzyme
GFM1	translation elongation Factor G 1, mitochondrial
GFM2	translation elongation Factor G 2, mitochondrial
GRPEL1	GrpE-Like 1 protein co-chaperone, mitochondrial
GRPEL2	GrpE-Like 2 protein co-chaperone, mitochondrial
GRSF1	G-Rich RNA Sequence binding Factor 1
GroEL	Prokaryotic chaperonin Hsp60, peptide-dependent ATPase
GSA	Glutamate γ-Semialdehyde
GSAM	Glutamate-1-Semialdehyde-2,1-AminoMutase
GTP	Guanosine 5’-Triphosphate
GTPase	Guanosine 5’-Triphosphatase
HARS2	Histidine-tRNA synthase, mitochondrial
HemL	Prokaryotic ortholog of GSAM, heme/porphyrin biosynthesis enzyme
HPLC	High-performance liquid chromatography
Hsp100	Eukaryotic heat shock protein family, ~100 kDa size in yeast
HSP60	Chaperonin family of heat shock proteins, ~60 kDa size in bacteria
Hsp70	Ubiquitous family of heat shock proteins, homologous to DnaK
HSP90	Bacterial/eukaryotic family of heat shock proteins
HSPA9	Mortalin/GRP75, heat shock protein family A (Hsp70) member 9, mitochondrial
HssR	Prokaryotic Heme response Regulator
IBAQ	Intensity-Based Absolute Quantification
ICP-MS	Inductively coupled plasma mass spectrometry
ID	Identity code
IF2	Prokaryotic translation Initiation Factor 2, protects formylMet-tRNA
InfB	Prokaryotic translation Initiation Factor if-2
IRP1	IREB1/Aconitase-1, Iron Regulatory Protein 1
kDa	kilodalton
KGD4	MRPS36, Alpha-Ketoglutarate Dehydrogenase Component 4
LARS2	Leucine-tRNA synthase, mitochondrial
LC/MS	Liquid Chromatography/Mass Spectrometry
LepA	Prokaryotic elongation factor, back-translocating, stress fidelity, GTPase
LONP1	Lon Peptidase 1, Mitochondrial
LRPPRC	Leucine Rich Pentatricopeptide Repeat Containing
MCIA	Mitochondrial Complex I Assembly complex
MDa	Megadalton
MEF	Murine Embryonic Fibroblasts
MICOS	Mitochondrial Contact site and cristae Organizing System
MIDAS	Metal Ion-Dependent Adhesion Site
Mg2+	Magnesium ion
mM	millimolar
MoxR	Prokaryotic magnesium chelatase, AAA+ ATPase
MRPL18	Mitochondrial Ribosomal Protein L18
MRPL38	Mitochondrial Ribosomal Protein L38
MRPP1	=TRMT10C, Mitochondrial Ribonuclease P Protein 1
MS	Mass Spectrometry
MSMS	tandem Mass Spectrometry
MT-ATP8	Mitochondrially encoded ATP synthase membrane subunit 8
MTCO1	COX1, Mitochondrially encoded Cytochrome c Oxidase I
MTCO2	COX2, Mitochondrially encoded Cytochrome c Oxidase II
MTCO3	COX3, Mitochondrially encoded Cytochrome c Oxidase III
MTHFD2	Methylenetetrahydrofolate Dehydrogenase (NADP+ dependent) 2
mtLSU	mitochondrial Large Subunit of ribosome
MTND3	Mitochondrially encoded NADH Dehydrogenase 3
MTND4	Mitochondrially encoded NADH Dehydrogenase 4
MTND5	Mitochondrially encoded NADH Dehydrogenase 5
MTND6	Mitochondrially encoded NADH Dehydrogenase 6
mtSSU	mitochondrial Small Subunit of ribosome
*m*/*z*	ratio of mass versus charge number of ions
NaCl	Sodium Chloride salt
NADH	reduced form of Nicotinamide Adenine Dinucleotide
NDUFAB1	mt-ACP2, NADH:Ubiquinone oxidoreductase subunit AB1
NDUFAF1	NADH:Ubiquinone oxidoreductase complex Assembly Factor 1
NDUFAF3	NADH:Ubiquinone oxidoreductase complex Assembly Factor 3
NDUFS1	NADH:Ubiquinone oxidoreductase core subunit S1
NDUFV1	NADH:Ubiquinone oxidoreductase core subunit V1
NDUFV2	NADH:Ubiquinone oxidoreductase core subunit V2
NP40	Nonyl Phenoxypolyethoxylethanol, non-ionic, non-denaturing detergent
NPET	Nascent Polypeptide Exit Tunnel in large subunit of ribosome
NTPase	Nucleoside-Triphosphatase
OAT	Ornithine delta-Aminotransferase, mitochondrial
p	Probability of error
P21	Postnatal day 21
P5C	Pyrroline-5-Carboxylate
P5CS	ALDH18A1, Pyrroline-5-Carboxylate Synthetase
PARL	Presenilin-Associated-Rhomboid-Like, mitochondrial intramembrane protease
PBS	Phosphate-buffered saline solution
PBS/T	Phosphate-buffered saline solution with Tween 20
PCR	Polymerase chain reaction
PDK1	Pyruvate Dehydrogenase Kinase 1
PDK3	Pyruvate Dehydrogenase Kinase 3
PDPR	Pyruvate Dehydrogenase Phosphatase Regulatory subunit
pH	“potential of Hydrogen”, scale to quantify acidity of aqueous solutions
PHB1	Prohibitin-1, forms large ring complexes in mitochondrial inner membrane
PLP	P5P, Pyridoxal 5’-Phosphate, vitamin B6 active form, enzyme cofactor
PLPBP	PROSC, Pyridoxal Phosphate Binding Protein
POLDIP2	DNA Polymerase Delta Interacting Protein 2
PP/PE	Polypropylene/Polyethylene
PPIX	Protoporphyrin IX
PPOX	Protoporphyrin IX oxidase
PRIDE	Proteomics Identification Database—EMBL/EBI
PRLTS3	Perrault Syndrome type 3
ProA	prokaryotic ortholog of ALDH18A1/P5CS
PRORP	MRPP3, Protein Only RNase-P Catalytic Subunit
PTCD1	Pentatricopeptide repeat Domain 1
PTCD2	Pentatricopeptide repeat Domain 2
PTCD3	MRPS39/COXPD51, Pentatricopeptide repeat Domain 3
RavA	Prokaryotic AAA+ moxr family ATPase, putative molecular chaperone
Rea1	Midasin-1, yeast chaperone for maturation of pre-60S ribosome
RF3	Prokaryotic translation Release Factor 3
RIPA	Radio-Immuno-Precipitation Assay buffer
RMND1	Required for Meiotic Nuclear Division 1 homolog
RNA	Ribo-Nucleic Acid
RpsA	Prokaryotic RNA chaperone to unfold structured mRNA on ribosome
rRNA	ribosomal RNA
RT	Room Temperature
RT-qPCR	Reverse Transcriptase real-time quantitative PCR
SC	Supercomplex
SDS	Sodium Dodecyl Sulfate, detergent
SEM	Standard Error of the Mean
SFXN4	Sideroflexin 4
SHMT2	Serine Hydroxy Methyltransferase 2
SLC25A28	Solute Carrier family 25 member 28, mitochondrial iron transporter
SLIRP	SRA Stem-Loop Interacting RNA-binding Protein, mitochondrial
SLP2	STOML2, Stomatin-Like Protein 2, at inner membrane rafts
SPATA5	Spermatogenesis-associated factor protein, mitochondrial ribosome maturation
SPG7	Matrix-AAA peptidase subunit, paraplegin
STRING	Search Tool for the Retrieval of Interacting genes/Proteins
T	statistical trend
*Tbp*	TATA-Binding Protein
TBRG4	FASTKD4, Transforming growth factor Beta Regulator 4
TIMMDC1	Translocase of Inner Mitochondrial Membrane Domain Containing 1
TMEM126B	Transmembrane protein 126B, mitochondrial complex I assembly factor
TMEM186	Transmembrane protein 186, mitochondrial complex I assembly factor
TRAP1	HSP90L, TNF Receptor Associated Protein 1, mitochondrial chaperone
TRMT10C	MRPP1, tRNA Methyltransferase 10C, mitochondrial RNase-P Subunit
tRNA	transfer RNA
tRNAPhe	tRNA for the amino acid Phenylalanine
tRNAVal	tRNA for the amino acid Valine
TUBA4A	Tubulin Alpha 4a protein
TWNK	Twinkle mtDNA helicase, Ataxin-8
UPR	Unfolded Protein Response pathway
UPRmt	Unfolded Protein Response in mitochondria
*v*/*v*	ratio volume per volume
VCP	Valosin Containing Protein, transitional ER AAA+ ATPase
ViaA	Prokaryotic VWA-domain-protein, CoxE-like family, AAA+ interacting
VWA	von Willebrand factor type A domain
VWA8	Von Willebrand factor A domain containing 8
WT	wildtype
*w*/*v*	ratio weight per volume
YME1L1	ATP-dependent zinc metalloprotease, mitochondrial intermembrane space
ZFE	Central Animal Facility, University Hospital Frankfurt am Main
