# Supplementary material for: Translation Fidelity and Respiration Deficits in CLPP-Deficient Tissues: Mechanistic Insights from Mitochondrial Complexome Profiling
Source: Int J Mol Sci. 2023 Dec 15;24(24):17503. doi: 10.3390/ijms242417503 (PMC10743472; doi:10.3390/ijms242417503)
Supplement: Supplementary file 1 [file ijms-24-17503-s001.zip › supplementary.pdf]

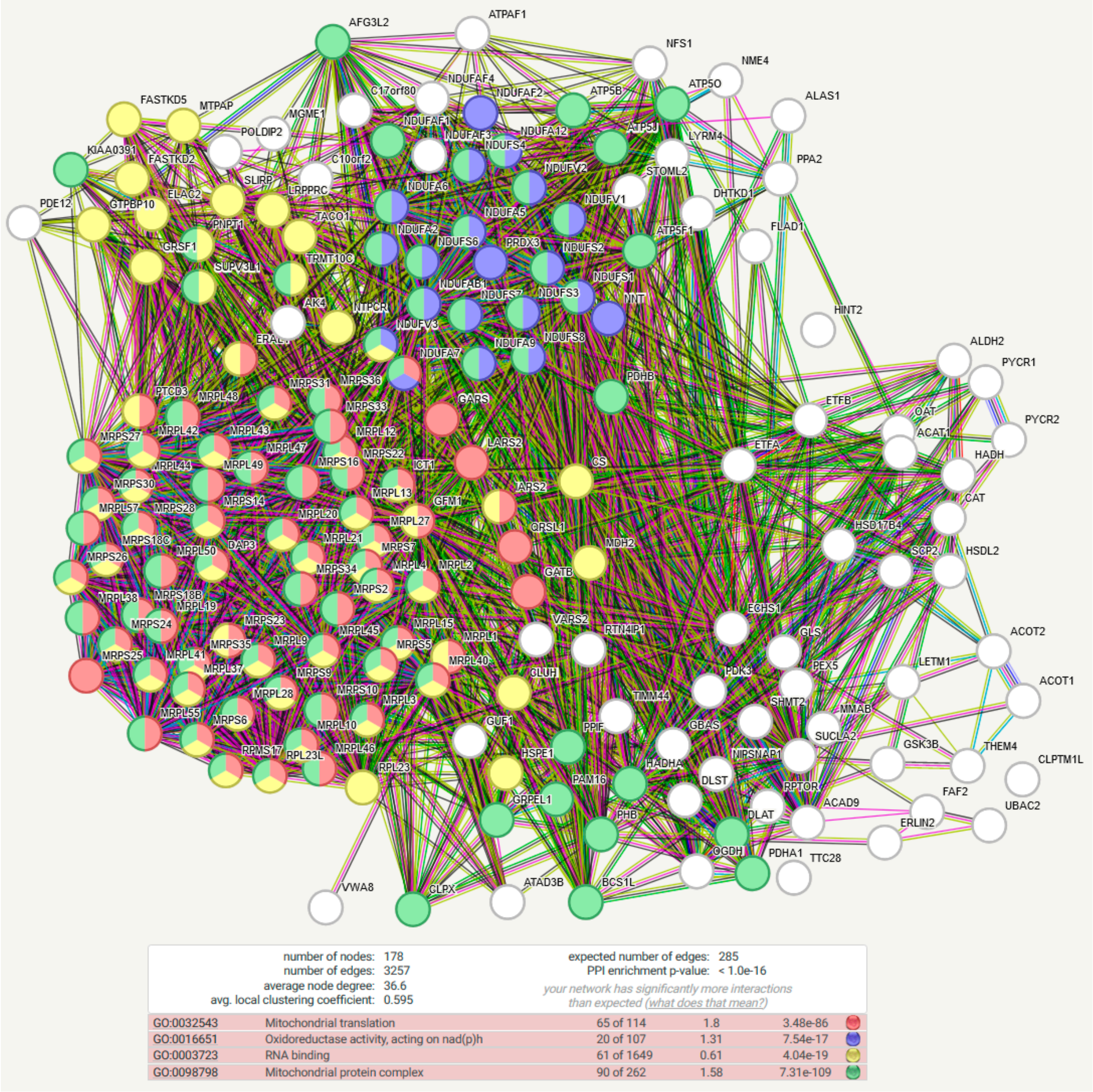

Figure S1. VWA8 as prey in human protein-protein-interactions, according to BIOGRID database, visualized and analyzed for pathway enrichments by the STRING webserver. To avoid overexpression artifacts where excess VWA8 remains excluded from mitochondrial import and interacts with cytosolic or nuclear factors, only the BIOGRID dataset on VWA8 as prey was taken for analysis, not the dataset with VWA8 as bait. Practically all interactors are known for their localization to the mitochondrial matrix or inner membrane. Their overall protein-protein-interaction enrichment is highly significant (<1.0e-16), as illustrated by lines that highlight interactions previously known to STRING in several colors (each color representing different technical approaches of ascertainment and credibility). The interacting proteins are illustrated as buttons where different colors reflect their involvement in assembly complexes (green color) or pathways, e.g. the mitoribosomal translation apparatus (red), the respiratory chain complexes I-V (blue), RNA-binding proteins many of which cluster in the mitochondrial RNA granule (yellow). The significance of enrichment is analyzed in lines at the lower figure end. In columns, the webserver shows for each Gene Ontology (GO) term (1) the number, (2) the description, (3) the count of factors observed among all factors in the network, (4) the enrichment strength, (5) the false discovery rate (FDR). As shown, the BIOGRID database comprises VWA8 interaction data well beyond previous STRING interaction knowledge, and indicates the association of the VWA8 unfoldase with the mitochondrial RNA granule, the mitoribosomal translation machinery, several respiratory chain complexes, and various metabolic enzymes.

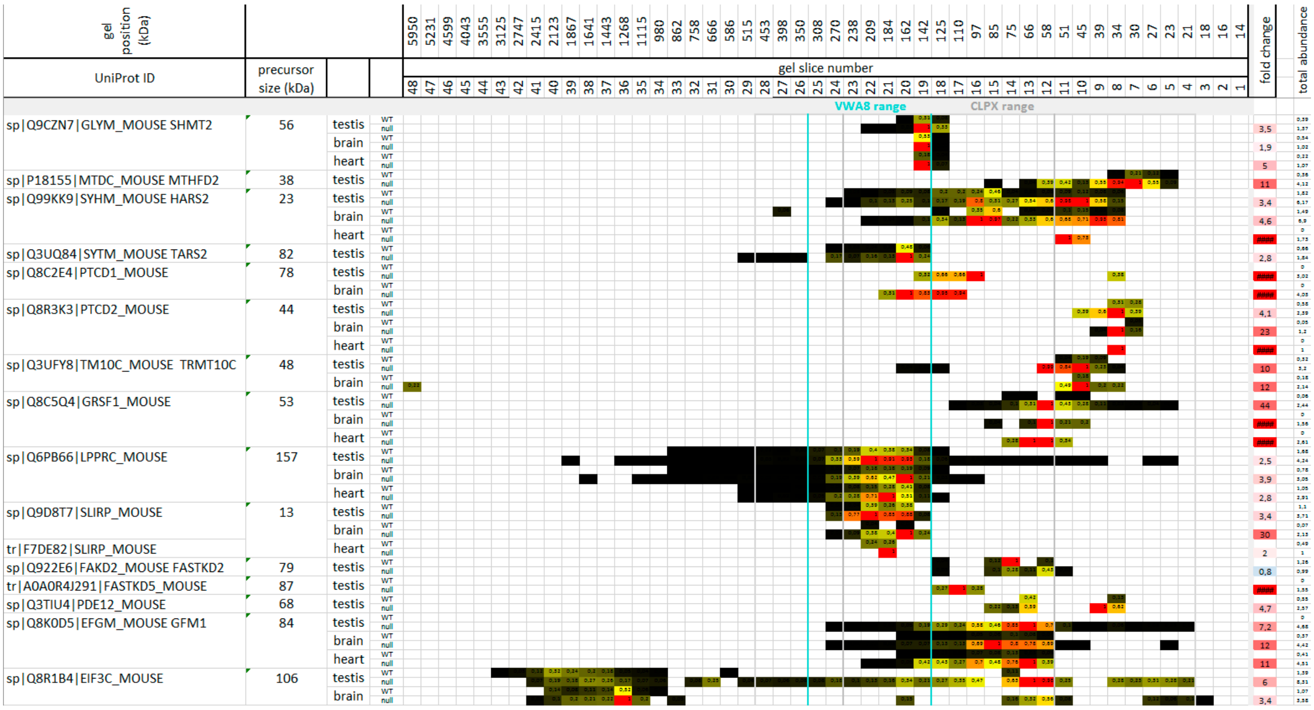

Figure S2. Complexomics profiles of ribonucleoproteins from the mitochondrial matrix that showed CLPX/VWA8 comigration/dispersion as well as accumulated abundance in CLPP-null tissues. The format is analogous to Figure 2. Overall, the physiological CLPX and VWA8 migration range (dark grey box) overlaps with several mRNA translation factors (GFM1, EIF3C, HARS2, TARS2), while the dispersed CLPX (light grey box) and VWA8 migration range also includes various RNA processing factors (LRPPRC, SLIRP, MTHFD2, PTCD1, PTCD2, TRMT10C/MRPP1, GRSF1, FASTKD2, PDE12).

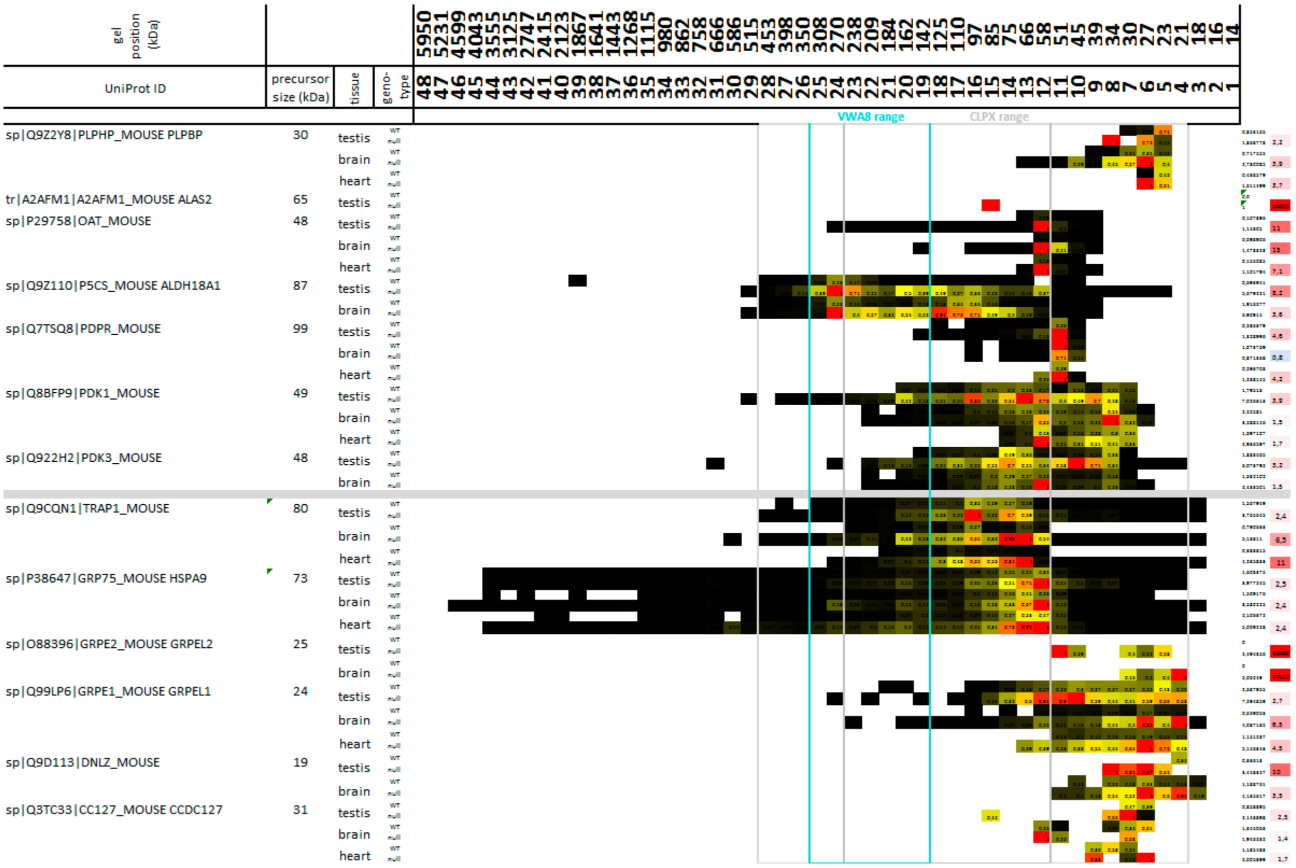

Figure S3. Complexomics profiles of mitochondrial matrix PLP-associated factors and other chaperones that showed CLPX/VWA8 comigration/dispersion as well as accumulated abundance in CLPP-null tissues. The format is analogous to Figure 2. Overall, the dispersed CLPX (light grey box) migration range overlaps with PLP-associated proteins (PLPBP, ALAS2, OAT) and with several other chaperone-pathway factors, but also with unrelated pyruvate-homeostasis enzymes.

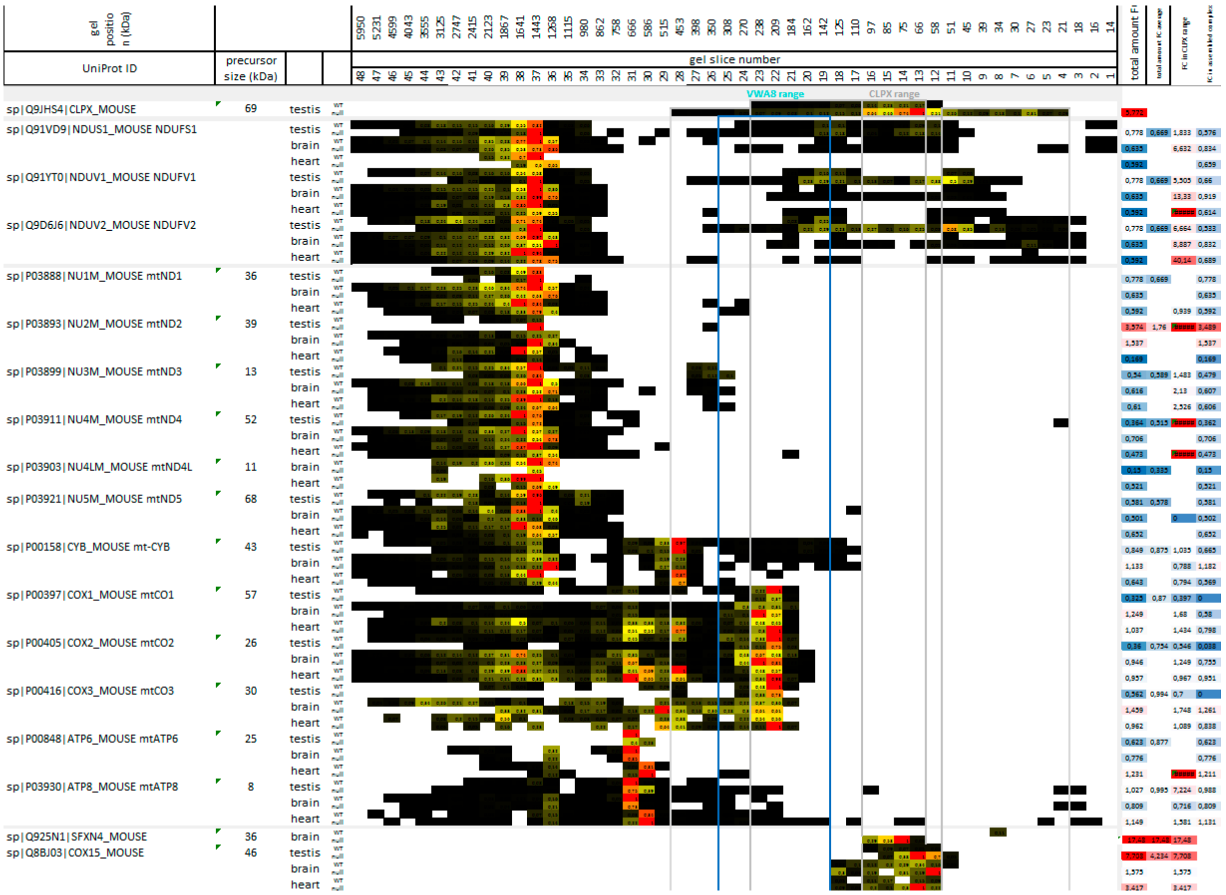

Figure S4. Complexomics profiles of the detected mitochondrially translated OXPHOS complex components and relevant assembly factors, regarding CLPX/VWA8 comigration/dispersion as well as accumulated abundance in CLPP-null tissues. Three matrix-immersed complex-I N-module core factors (S1, V1 and V2) that were shown to depend on CLPP in their assem-bly/disassembly, and the 12 detected factors that are mitochondrially encoded/translated within respiratory chain complexes I/III/IV/V were studied. Fold changes were calculated (1) across all slices per tissue (total amount), (2) as average of tissues, (3) in the CLPX migration range from slice 4 to 28, (4) in the fully assembled complex from slice 29 to 48. The color code is analogous to Figure 2. Comigration with CLPX was detected mainly for the N-module factors and for MTCO1-3 (COX1-3), but none of them showed accumulation upon CLPP deficiency. The N-module factors accumulated selectively within the CLPX comigration range, while MTCO1-3 were reduced also there. Integration in fully assembled complexes was reduced for N-module factors and for almost all mitochondrially encoded proteins, reaching values of almost 0 for MTCO1-3. Among complex-IV components, COX15 was conspicuous for its comigration with CLPX and its strong accumulation in CLPP-null tissues. When individual factors due to scarce abundance were not detectable at all by mass spectrometry in a WT sample, the resulting FC values of infinite upregulation in mutant can be considered artefacts.
