# Supplementary figures and images for: Translation Fidelity and Respiration Deficits in CLPP-Deficient Tissues: Mechanistic Insights from Mitochondrial Complexome Profiling
Source: Int J Mol Sci. 2023 Dec 15;24(24):17503. doi: 10.3390/ijms242417503 (PMC10743472; doi:10.3390/ijms242417503)

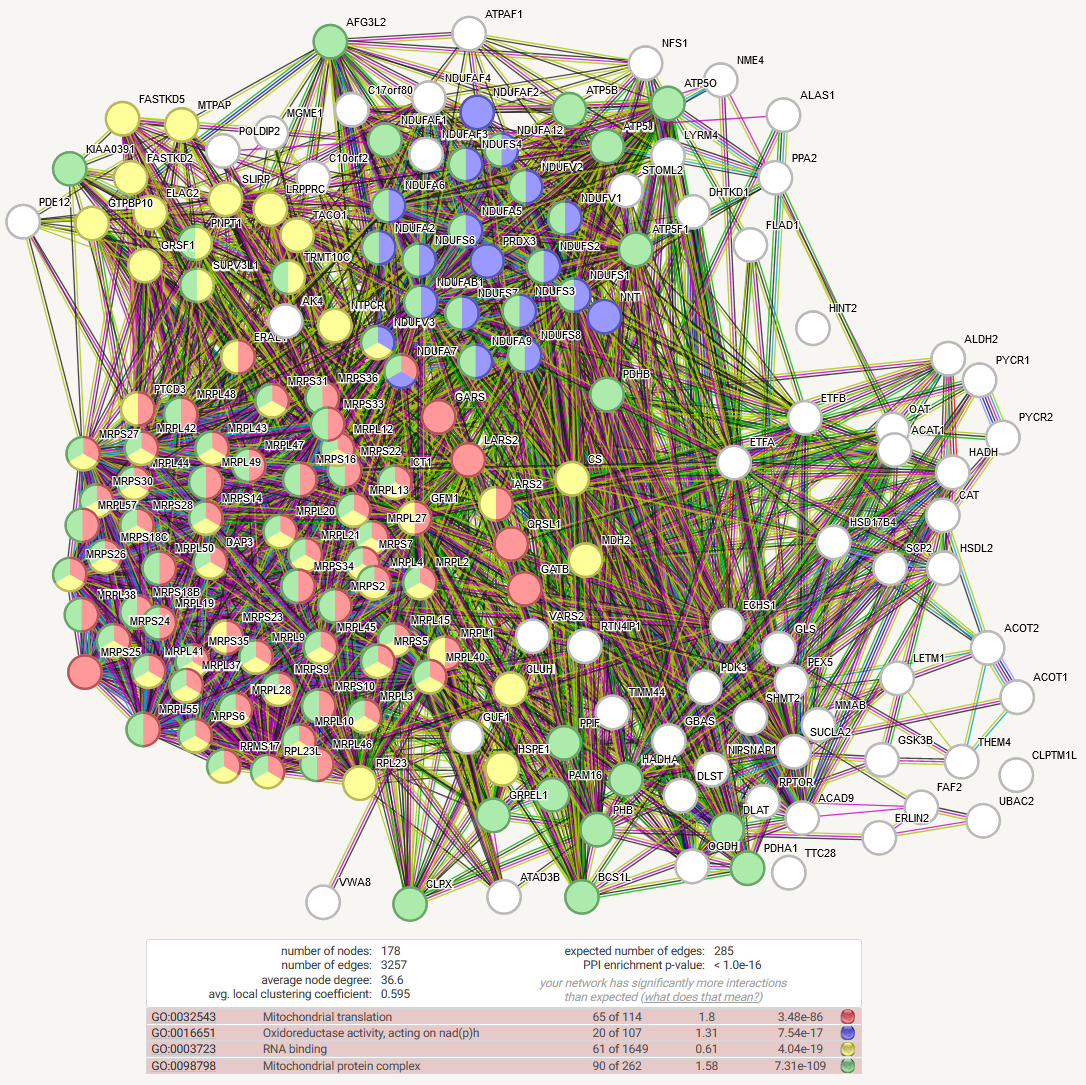

Supplement: Supplementary file 1 [file ijms-24-17503-s001.zip › Figure S1-VWA8human-InteractingPreysBIOGRID.tif]

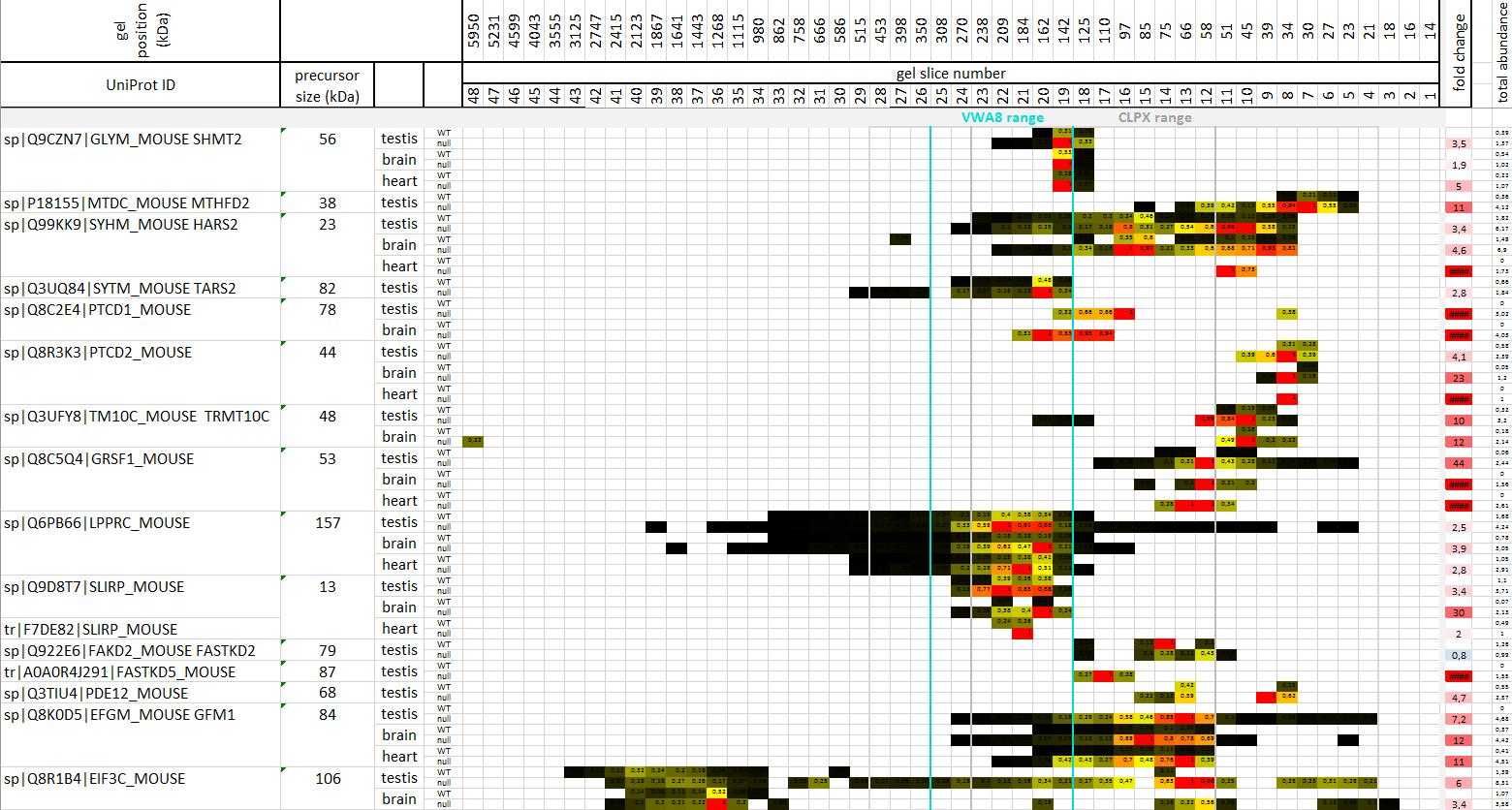

Supplement: Supplementary file 1 [file ijms-24-17503-s001.zip › Figure S2-CLPX-VWA8-ComigratingDisperseAccumulated-Ribonucleoproteins-TestisBrainHeart-quantitative.tif]

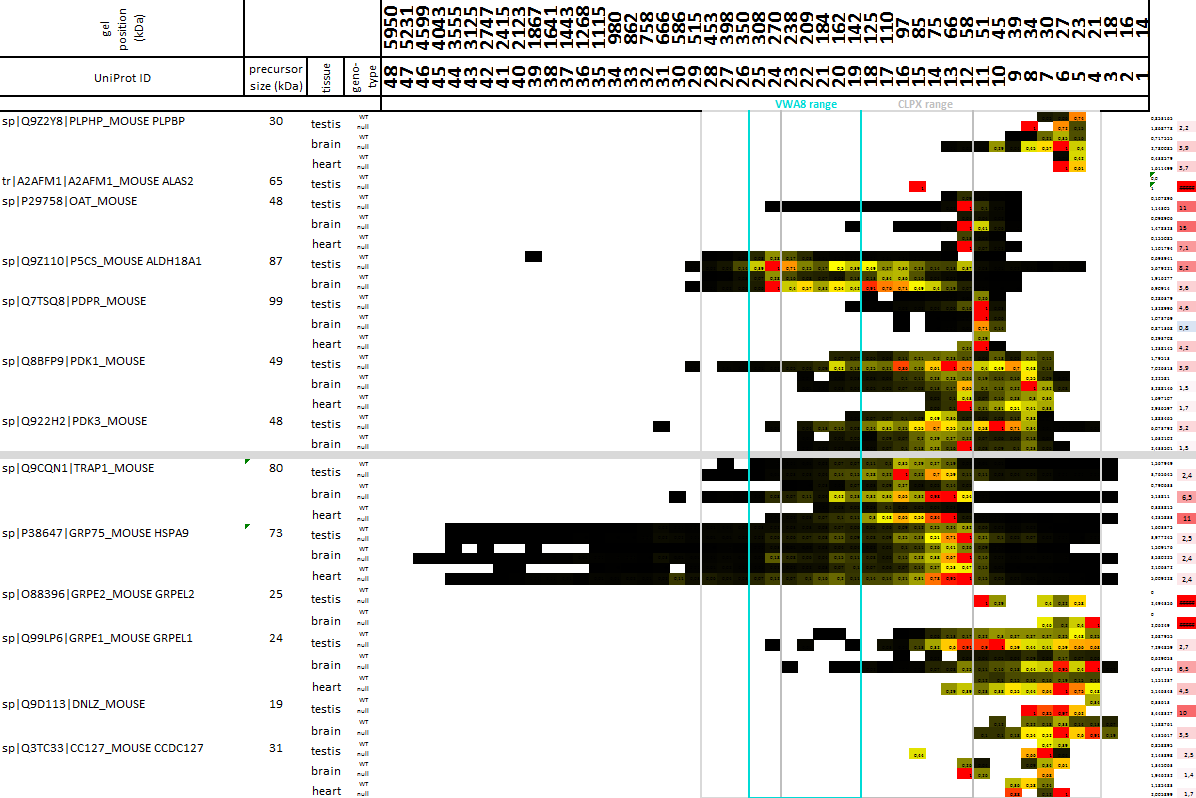

Supplement: Supplementary file 1 [file ijms-24-17503-s001.zip › Figure S3-CLPX-VWA8-ComigratingDisperseAccumulated-PLPfactors-ChaperonesTestisBrainHeart_quantitative.tif]

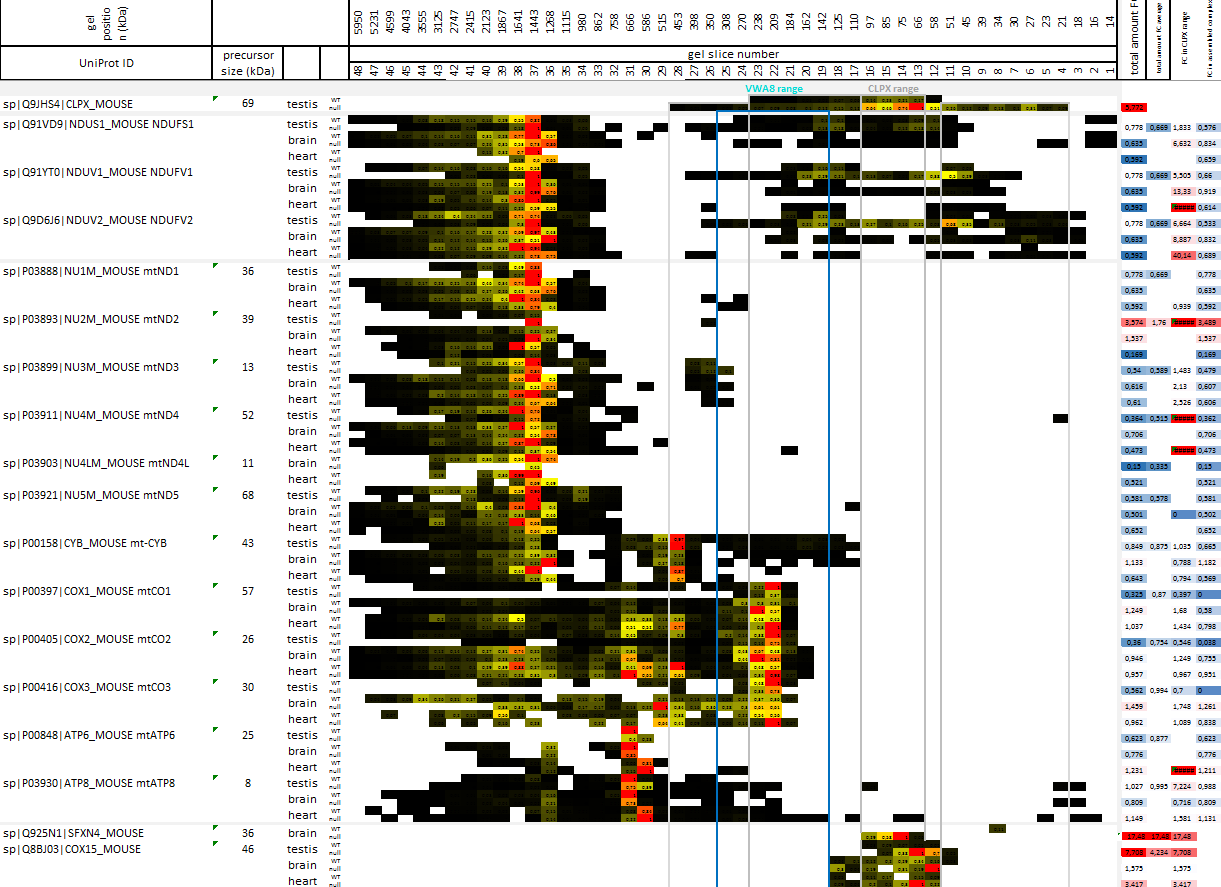

Supplement: Supplementary file 1 [file ijms-24-17503-s001.zip › Figure S4-CLPX-VWA8-ComigratingDisperseAccumulated_mtOXPHOS_TestisBrainHeart_quantitative.tif]
